# Interface Design for Stretchable Electronic Devices

**DOI:** 10.1002/advs.202004170

**Published:** 2021-02-22

**Authors:** Dong Wook Kim, Minsik Kong, Unyong Jeong

**Affiliations:** ^1^ Department of Materials Science and Engineering Pohang University of Science and Technology (POSTECH) 77 Cheongam‐Ro, Nam‐Gu Pohang Gyeongbuk 37673 Republic of Korea

**Keywords:** device fabrication, interface design, stretchable electronics, stretchable materials

## Abstract

Stretchable electronics has emerged over the past decade and is now expected to bring form factor‐free innovation in the next‐generation electronic devices. Stretchable devices have evolved with the synthesis of new soft materials and new device architectures that require significant deformability while maintaining the high device performance of the conventional rigid devices. As the mismatch in the mechanical stiffness between materials, layers, and device units is the major challenge for stretchable electronics, interface control in varying scales determines the device characteristics and the level of stretchability. This article reviews the recent advances in interface control for stretchable electronic devices. It summarizes the design principles and covers the representative approaches for solving the technological issues related to interfaces at different scales: i) nano‐ and microscale interfaces between materials, ii) mesoscale interfaces between layers or microstructures, and iii) macroscale interfaces between unit devices, substrates, or electrical connections. The last section discusses the current issues and future challenges of the interfaces for stretchable devices.

## Graphical abstract

Stretchable electronic devices have diverse interfaces that need to be considered in material design and device fabrication. This article reviews representative approaches to solve the technological issues in stretchable electronic devices, focusing on the interfacial design in different scales (nano/microscale, mesoscale, macroscale). It also discusses the technological advances required for future stretchable device development.

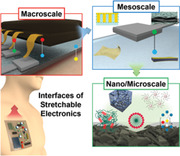

## 1 Introduction

Over the past decade, stretchable electronics have received intensive attention due to the possibility of taking new device form factor and finding new applications that cannot be achieved with the rigid conventional devices.^[^ ^1^, ^2^, ^3^ ^]^ The studies for achieving stretchability have been performed in the aspects of new material developments,^[^ ^1^, ^4^ ^]^ strain engineering,^[^ ^5^, ^6^ ^]^ and testing practical applications.^[^ ^7^, ^8^ ^]^ Those efforts have brought great advances in fabrication of stretchable device units, which include electrodes,^[^ ^9^, ^10^, ^11^ ^]^ interconnections,^[^ ^12^, ^13^, ^14^ ^]^ light‐emitting layers,^[^ ^15^, ^16^, ^17^ ^]^ transistors,^[^ ^18^, ^19^, ^20^ ^]^ batteries,^[^ ^21^, ^22^, ^23^ ^]^ and supercapacitors.^[^ ^24^, ^25^, ^26^ ^]^ In recent years, a variety of complete stretchable devices have been developed.^[^ ^27^, ^28^, ^29^, ^30^, ^31^, ^32^, ^33^, ^34^, ^35^, ^36^, ^37^, ^38^, ^39^, ^40^, ^41^ ^]^ Pixelated physical sensors showing the distribution of applied pressure and strain have been demonstrated for the uses as electronic skins (E‐skins),^[^ ^27^, ^28^, ^29^ ^]^ artificial skins for robots,^[^ ^30^, ^31^, ^32^ ^]^ wearable Braille readers,^[^ ^33^ ^]^ and skin‐attachable biosensors.^[^ ^34^, ^35^ ^]^ The stretchable physical sensors have also been used as implantable medical sensors for monitoring the movement of organs in real time.^[^ ^36^, ^37^ ^]^ Recently, the stretchable devices extended their applications to chemical sensing^[^ ^38^ ^]^ and bioelectrical signal sensing.^[^ ^39^ ^]^ The noninvasive glucose sensors integrated in smart contact lens and smart patches are representative examples of the stretchable chemical sensors.^[^ ^40^ ^]^ The stretchable patterned electrode wrapping the nerve fibers has been used for electrical stimulation and also for signal detection in the brain.^[^ ^41^ ^]^

A usual approach of manufacturing a stretchable device is to assemble an intrinsically stretchable active layer on a stretchable electrode,^[^ ^42^, ^43^ ^]^ then interconnect the active unit to other device units through a stretchable conducting line.^[^ ^44^, ^45^ ^]^ In this approach, all the device components are stretchable. Another approach is to design the substrate so that the strain is concentrated in the interconnections, and there is no (or limited) strain in the unit devices.^[^ ^46^, ^47^ ^]^ This approach allows the use of rigid unit devices. Both approaches have many interfaces requesting different scales and properties, hence design of the interfaces determines the device characteristics and the level of stretchability. As electrically and optically active materials are usually not stretchable, in order to give stretchability to them as in the first approach, uniform mixing with elastic materials (usually polymers)^[^ ^48^, ^49^ ^]^ or controlling their microphase separation in an elastic matrix^[^ ^50^, ^51^ ^]^ is often used. Thus, the design of nanoscale interfaces between molecules or between morphological microstructures is important to obtain stretchability. A unit device consists of multiple active layers and contains several interfaces in mesoscale (tens of micrometers). Mechanical deformation on the mesoscale interfaces causes cracks, wrinkles, crazes, and structural changes that are determined by the difference in the mechanical properties across the interface.^[^ ^52^, ^53^ ^]^ Control of the mesoscale interfaces is typically related with the fabrication process, which is important in securing the reliability and reproducibility of the device. Once the unit devices are fabricated, understanding the strain distribution in macroscale and reducing the strain effect at the interfaces between the unit devices and the interconnections are critical to avoid the morphological defects and to achieve long‐term durability of the device.^[^ ^54^, ^55^ ^]^

**Figure 1:**
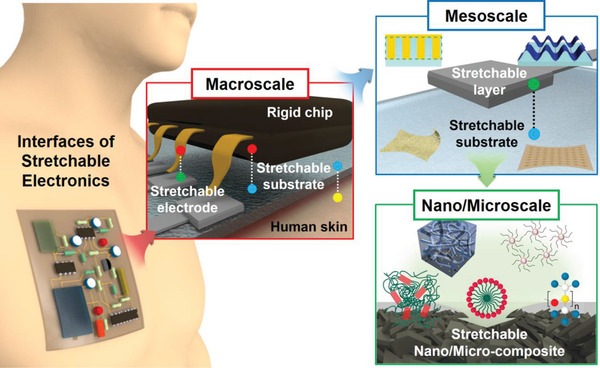
Interface design in different scales for stretchable electronic devices.

**Figure 2:**
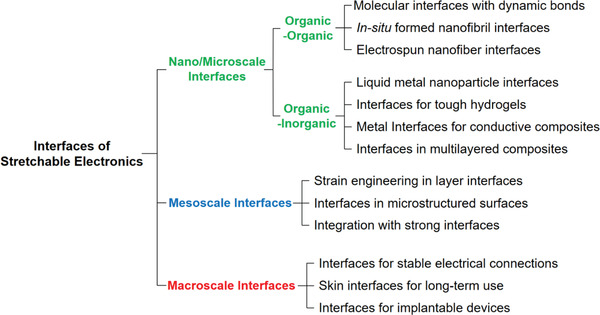
Classification of interfaces in stretchable electronic devices.

In this article, we review some recent advances in stretchable electronic devices, focusing on the design of the interfaces in different scales (**Figures** **1** and **2**). Sections 2 and 3 cover the nano‐ and microscale interfaces by dividing into organic/organic interfaces (Section 2) and organic/inorganic interfaces (Section 3). Section 4 deals with the mesoscale interfaces such as the contacts between two layers or relatively large microstructures and microphases. Section 5 presents the macroscale interfaces between the unit devices, substrates, and interconnections. The last section discusses on the technological advances to be achieved and suggests possible ways for some advances.

## 2 Design of Nano/Microscale Organic–Organic Interfaces

Nano‐ and microscale organic–organic interfaces for obtaining stretchability have been studied in the following areas; control of the permanent or dynamic bonds between two molecules in a homogeneously mixed state,^[^ ^58^, ^59^, ^60^ ^]^ control of the dynamic bonding in the functional nanodomains where microphase separation is present,^[^ ^63^, ^64^ ^]^ adjusting the dynamic interaction between the functional nanodomains and the matrix,^[^ ^67^, ^68^, ^69^, ^70^, ^71^ ^]^ creating strong interface between the organic matrix and the nanofibrils which is advantageous for securing stretchability due to its long contour path,^[^ ^62^, ^73^, ^74^, ^75^, ^76^, ^77^, ^78^ ^]^ and adjusting the interaction between infinitely long organic nanofibers and the organic matrix.^[^ ^82^, ^83^, ^84^ ^]^ This section introduces some outstanding works for the different interfacial aspects.

### 2.1 Dynamic Bond Molecular Interfaces

Stretchable devices require reversible elasticity in a large tensile strain range (typically, *ε* ≤ 100%). The reversible stretchability of the entire device comes from the elastomeric substrate because the retractive force of the substrate can enforce the viscoelastic components to retain the initial dimension. Initially, covalent‐bonded network polymers such as poly(dimethylsiloxane) (PDMS) and polyurethane elastomers have been widely used.^[^ ^56^, ^57^ ^]^ Recently, new substrates were developed to attain novel functionality in the substrate. Representative approaches for the functional substrates are based on the dynamic bonding interface, that is, physical crosslinking between the molecules, instead of the permanent covalent crosslinking. The dynamic interactions are based on the hydrogen bonding,^[^ ^58^, ^59^, ^60^, ^67^ ^]^ the metal coordination,^[^ ^63^, ^64^, ^68^ ^]^ and the host–guest interaction^[^ ^69^, ^70^, ^71^ ^]^ that are repeatedly broken and healed. The following are the representative achievements for those approaches.

**Figure 3:**
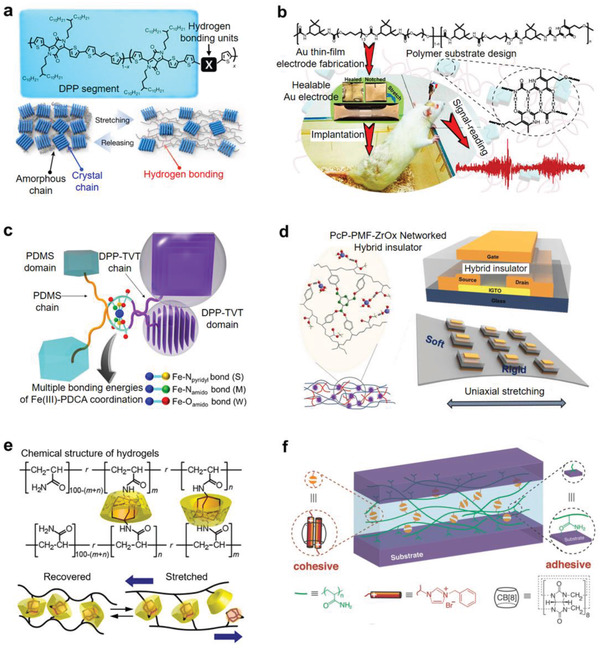
Dynamic bond interfaces a) for a stretchable substrate by hydrogen bonding, b) for dielectric by metal coordination, for semiconductors by c) hydrogen bonding and d) metal coordination, and e,f) for substrates by host–guest interaction. a) Supramolecular block copolymer substrate with multiple intra‐ and interchain hydrogen bonds and its use in the stretchable and self‐healable Au film electrode. Reproduced with permission.^[^ ^58^ ^]^ Copyright 2018, American Chemical Society. b) Zr coordination‐based stretchable dielectric insulator with reliable viscoelasticity and high insulating performance. Reproduced with permission.^[^ ^64^ ^]^ Copyright 2020, Wiley‐VCH. c) Hydrogen bonding units in the stretchable semiconducting polymer backbone to promote dynamic crosslinking of the conjugated semiconducting polymers. Reproduced with permission.^[^ ^67^ ^]^ Copyright 2016, Springer Nature. d) Dynamic metal–ligand coordination between semiconducting polymers and elastomer chains for the stretchable, tough, and self‐healable semiconductor. Reproduced with permission.^[^ ^68^ ^]^ Copyright 2019, AAAS. e) Dynamic host–guest interaction in the supramolecular hydrogels with reversible stretchability and high toughness. Reproduced with permission.^[^ ^69^ ^]^ Copyright 2013, American Chemical Society. f) CB[8] Supramoleculer gel based on the host–guest interaction used as an adhesive for two substrates. Reproduced with permission.^[^ ^71^ ^]^ Copyright 2018, Wiley‐VCH.

Yan et al. developed a supramolecular block copolymer substrate which has multiple intra‐ and interchain hydrogen bonds in the microphase‐separated nanosized domains (**Figure** **3**a).^[^ ^58^ ^]^ The dynamic bonding nature provided extremely high stretchability (*ε* ≈ 17 000%) and self‐healed the scratches and cuts made on the substrate within 48 h at room temperature. The Au film deposited on the supramolecular polymer substrate was electrically conductive even at a large strain (*ε* = 400%). Surprisingly, the Au film was self‐healable, which was attributed to the improved interfacial adhesion and the energy dissipation of the substrate while being stretched. The Au film electrodes were attached to human skin and implanted in a live rat for obtaining the electromyography (EMG) signals. Similarly, various elastic substrates with dynamic hydrogen bonds were developed for the stretchable and self‐healable electrodes in E‐skins. Hao et al. inserted low‐molecular‐weight glycerol in hydroxyethylcellulose to create hydrogen bonds between the polymer chains, and they formed a macromolecular gel with a high stretchability (*ε* ≈ 300%) and the self‐healing performance.^[^ ^59^ ^]^ Kang et al. reported a supramolecular polymer film constructed with a network of crosslinked polymer chains having both strong and weak hydrogen bonding units.^[^ ^60^ ^]^ The strong hydrogen bonding units formed elastic nanodomains, and the weak bonding units dissipated the energy through the breakage of the bonds. The two units contributed to the high stretchability (*ε* = 1200%) and high toughness (12 000 J m^−2^) of the film. The supramolecular films combined with single‐wall carbon nanotubes (SWCNTs)^[^ ^59^ ^]^ and liquid metal^[^ ^60^ ^]^ were also used for the stretchable and self‐healable electrode.

Practical electronic devices require a good dielectric material. Relatively, stretchable high‐performance dielectric materials have not been investigated, thus the insulating stretchable substrates such as PDMS^[^ ^61^ ^]^ and polystyrene‐*block*‐poly(ethylene butylene)‐*block*‐polystyrene (SEBS) block copolymer^[^ ^62^ ^]^ have been used. Recent studies have made a few advances on the basis of the molecular interface control. Rao et al. developed a stretchable and self‐healable dielectric elastomer by introducing metal–ligand coordination as the crosslinking site of the PDMS polymer chains.^[^ ^63^ ^]^ Different interactions between the bipyridine ligands and the various transition metal salts (FeCl_2_, Fe(BF_4_)_2_, ZnCl_2_, Zn(OTf)_2_, and Zn(ClO_4_)_2_) influenced the mechanical and electrical properties of the dielectric. For instance, a PDMS substrate crosslinked by the ZnCl_2_–bipyridine coordination demonstrated a high stretchability (*ε* = 300%) and self‐healing ability (taking 48 h at room temperature), as well as an enhanced dielectic property (a 1.2‐fold increase in the dielectric constant compared to the pure bipyridyl‐bridged PDMS). Kim et al. developed a metal coordination‐based stretchable dielectric material which has reliable viscoelasticity and high insulating performance (Figure 3b).^[^ ^64^ ^]^ Zirconia (Zr) coordination units with high electric permittivity crosslinked dynamically the poly(4‐vinylphenol‐*co*‐methylmethacrylate) (PcP) backbone chains and the crosslinkers with different binding structures (branched, linear, network). The dielectric film crosslinked with the linear‐structured crosslinkers (1,6‐bis(trimethoxysilyl)hexane) (BTMSH) exhibited the lowest leakage current, high permittivity, and good mechanical deformability. Such high performance was obtained because BTMSH has excellent chemical reactivity to the PcP backbone (Ph—O—Si) and ZrO*_x_* (Zr—O—Si). The leakage current and the relative dielectric constant of the hybrid dielectric film with BTMSH and ZrO*_x_* were improved substantially from 9.1 × 10^−4^ A cm^−2^ and 5.1 (for pristine PcP film) to 1.8 × 10^−8^ A cm^−2^ and 5.2, respectively.^[^ ^64^ ^]^

The conventional semiconductor society has believed that a high‐quality crystal without amorphous defects is prone to good performance semiconductors. Recent theoretical works anticipated that high mobility semiconductors can be obtained even in the amorphous state, and some experimental works proved it successfully.^[^ ^65^, ^66^ ^]^ To produce stretchable semiconductors, bridging crystalline nanodomains with stretchable amorphous matrix has been adopted. The dynamic bonding was used to produce stretchable semiconductor thin films. Oh et al. introduced the 2,6‐pyridine dicarboxamide (PDCA) hydrogen bonding units in the semiconducting polymer backbone to promote dynamic noncovalent crosslinking of the conjugated semiconducting polymers (Figure 3c).^[^ ^67^ ^]^ Upon tensile stretching, the amorphous nonconjugated PDCA crosslinking units dissipated the tensile stress through the breakage of hydrogen bonds, thus the crystalline semiconducting polymers could retain the charge transport mobility under stretching. This unique stress relaxation mechanism enabled the fabrication of stretchable transistors made of the semiconducting polymer. The transistor recovered its high field‐effect performance even after a hundred cycles of *ε* = 100% stretching. The same group introduced the dynamic metal–ligand coordination between the nanodomains of diketopyrrolopyrrole (DPP)‐based semiconducting polymer and the PDMS chains (Figure 3d).^[^ ^68^ ^]^ Both the PDMS and DPP semiconducting polymer backbones have PDCA moieties which can create Fe (III)‐PDCA dynamic coordination bonds. After broken, the bonds were spontaneously reconstructed, making brittle semiconducting polymer stretchable, tough, and self‐healable. The electrical performance of the DPP semiconductor/elastomer blend film was strain‐sensitive, depending on the percolation threshold of the DPP polymer. Thus, the semiconductor film was used as a variable strain sensing layer in a stretchable active‐matrix transistor sensor array for detecting strain distribution profiles.

The supramolecular hydrogels, which are crosslinked via the host–guest interactions, have been actively studied due to its high‐performance physical properties, such as high elasticity, toughness, and self‐healing characteristics.^[^ ^69^, ^70^, ^71^ ^]^ Kakuta et al. prepared the supramolecular hydrogel via host–guest interactions using *β*‐cyclodextrin (*β*CD) and adamantane (Ad) as the host and guest molecules, respectively (Figure 3e).^[^ ^69^ ^]^ The inclusion complexes of *β*CD‐acrylamide (AAm‐*β*CD) and Ad‐acrylamide (AAm‐Ad) acted as supramolecular crosslinkers, forming the stable cyclodextrin‐adamantane (*β*CD‐Ad) hydrogels. When the *β*CD‐Ad gel was stretched up to *ε* = 220%, the gel recovered its initial material strength (no residual strain) *ε* = 0%, whereas the chemically crosslinked poly(acrylamide) hydrogels did not show the reversible recovery. This was because when the gel was stretched below the yield strain, most of the inclusion complexes changed conformations and disassociated, but upon releasing the stress, the dissociated CD and Ad units autonomously reformed the initial complexes, and eventually restored the initial shape. Liu et al. demonstrated the supramolecular tough hydrogel networks via an in situ polymerization of acrylamide and functional guest molecules of (1‐benzyl‐3‐vinylimidazolium bromide), which were dynamically complexed with the cucurbit[8]uril (CB[8]) host molecules.^[^ ^70^ ^]^ The dynamic CB[8] host–guest complexes served as an effective energy dissipation components through the reversible association/dissociation, contributing to the high toughness and self‐healing property of these supramolecular hydrogel networks. The supramolecular gels with CB[8] host–guest complexes could be stretched more than 100 times of their original length and could complete self‐healing at room temperature. The same group reported that the CB[8]‐based suparmolecular hydrogels could serve as dynamic adhesives for diverse nonporous (glass and metal) and porous (wood and bone) substrates (Figure 3f).^[^ ^71^ ^]^ The dynamic CB[8] host–guest interactions inside the gel functioned as a cohesive promoter maintaining the gel toughness and reformable adhesion between two substrates, whereas the functional groups of the acrylamide backbone chains were physically adhered onto the substrates via Van der Waals interactions. This adhesive property of the supramolecular CB[8] hydrogel could be applied to the stretchable electronics or hybrid systems for biomedical devices or tissue/bone regeneration.

### 2.2 In Situ Formed Nanofibril Interfaces

**Figure 4:**
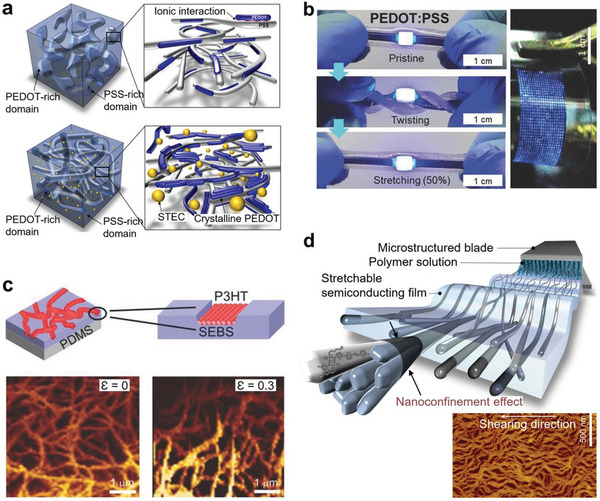
Nanofibril‐based stretchable electrodes and semiconductors. a) Incorporation of ionic liquids in PEDOT:PSS for highly conductive stretchable electrode. Reproduced with permission.^[^ ^73^ ^]^ Copyright 2017, AAAS. b) Addition of ionic surfactant (Triton X‐100) to PEDOT:PSS to produce viscoelastic PEDOT:PSS dough and its use as micropatternable stretchable electrodes.^[^ ^74^ ^]^ Copyright 2016, Wiley‐VCH. c) Network of P3HT nanofibril bundles on the surface on the SEBS matrix, generated by in situ phase separation of P3HT from SEBS. Reproduced with permission.^[^ ^77^ ^]^ Copyright 2015, Wiley‐VCH. d) Multiscale ordering and alignment of semiconducting nanofibrils in an elastomer matrix by the phase separation and shearing of the semiconducting polymer. Reproduced with permission.^[^ ^78^ ^]^ Copyright 2019, Springer Nature.

From the concept of the serpentine and buckling structures used for obtaining stretchability,^[^ ^72^ ^]^ nanosized fibers (nanofibrils) have been investigated to produce stretchable films of conductors and semiconductors. The concept requests in situ formation of the nanofibrils during the printing process and the good interfacial adhesion between the nanofibrils and the matrix. Coating of the preformed nanofibrils in the solution phase cannot produce uniform films in the entire area of the coated film. Poly(3,4‐ethylenedioxythiophene):poly(styrenesulfonate) (PEDOT:PSS) has been widely studied as a stretchable electrode based on the nanofibril concept. The interface could be controlled by the choice of additives. Wang et al. showed that ionic liquids incorporated in PEDOT:PSS resulted in the formation of the PEDOT nanofibrils upon spin‐coating (**Figure** **4**a).^[^ ^73^ ^]^ The ionic liquids transformed the structure of PEDOT into a crystalline fibrill so the conductivity of the PEDOT:PSS was enhanced. The long contour path of the curved nanofibrils provided stretchability. The ionic liquid served as a conductivity‐enhancing secondary dopant at the interface and also as a plasticizer to endow the stretchability in the PSS‐rich matrix. Various ionic liquids were tested to enhance the stretchability and electrical conductivity.^[^ ^73^ ^]^ PEDOT:PSS mixed with bis(trifluoromethane) sulfonimide lithium salt ionic liquid (>45%) was reported to have the highest stretchability (*ε* = 100%) and conductivity (>1000 S cm^−1^).^[^ ^73^ ^]^

Similar to ionic liquids, low‐molecular‐weight surfactants enhanced the conductivity and stretchability of the PEDOT:PSS. Oh et al. reported that an excess amount of a nonionic surfactant (Triton X‐100) added to PEDOT:PSS‐generated long PEDOT nanofibrils network in the PSS/surfactant matrix, and the entangled PEDOT fibril networks made PEDOT:PSS to be stretchable without conductivity degradation (Figure 4b).^[^ ^74^ ^]^ Adjusting the amount of the surfactant could control the viscoelasticity of the PEDOT:PSS like a rubber dough. The viscoelastic film became moldable into complex micropatterns and self‐healable by finger touch. The micropatterned PEDOT:PSS electrode was used to fabricate pixelated flexible organic light‐emitting diodes. High‐molecular‐weight water‐soluble polymers, such as poly(ethylene oxide) (PEO) and poly(vinyl alcohol) (PVA), could be used as effective additives to form the PEDOT nanofibrils for attaining stretchability.^[^ ^75^ ^]^ Addition of the adequate amount of the water‐soluble polymers enhanced the conductivity by screening the Coulombic interaction between the PEDOT nanofibrils and the PSS matrix. The stretchable PEO‐incorporated PEDOT:PSS electrode was used in fabricating the stretchable metal halide perovskite light‐emitting diodes.^[^ ^76^ ^]^

The nanofibril approach was also applied to the preparation of stretchable semiconductor films. Blending a conjugated semiconducting polymer with a viscoelastic polymer induced phase separation between the two polymers resulted in the formation of bundles of semiconductor nanofibrils. When the mixed solution of poly(3‐hexylthiophene) (P3HT) and SEBS block copolymer was spin‐coated, in situ phase separation of P3HT took place from the SEBS matrix and created the network of P3HT nanofibril bundles only on the surface of the SEBS matrix (Figure 4c).^[^ ^77^ ^]^ The P3HT/SEBS composite channel was used to fabricate the stretchable transistors which showed reliable performance up to 50% strain. Xu et al. successfully applied the same strategy to develop semiconducting polymer films with improved stretchability and charge transport mobility.^[^ ^62^ ^]^ Poly(2,5‐bis(2‐octyldodecyl)‐3,6‐di(thiophen‐2‐yl)diketopyrrolo[3,4‐c]‐pyrrole‐1,4‐dione‐*alt*‐thino[3,2‐b]thiophene) (DPPT‐TT) was selected as a high‐mobility semiconducting polymer and dispersed in the SEBS elastomer matrix. While the DPPT‐TT/SEBS solution was coated, the nanoconfinement effect suppressed the growth of large crystalline semiconducting nanofibers and formed the network of the aggregated DPPT‐TT nanofibrils in the SEBS matrix. Consequently, the on‐set strain for crack formation was increased, which allowed the fabrication of a stretchable semiconductor film with no cracks up to *ε* = 100%. The transistors made of the film exhibited a negligible decrease in mobility under harsh deformation, such as biaxial stretching (*ε*_x_ = *ε*_y_ = 100%), twisting, and poking. This so‐called “conjugated polymer/elastomer phase separation‐induced elasticity (CONPHINE)” methodology is acknowledged as one of the representative methods to fabricate the intrinsically stretchable transistor channels. The CONPHINE method was developed further into achieving multiscale ordering and alignment of the semiconducting nanofibrils in the elastomer matrix. By applying mechanical shearing to the semiconducting polymer/SEBS solutions using a line‐patterned microtrench blade, structurally ordered semiconducting nanofibrils were aligned to the shearing direction (Figure 4d).^[^ ^78^ ^]^ The aligned conjugated polymer nanofibrils promoted short‐range *π*–*π* ordering and reduced the energetic barrier for charge transport. As a result, the mobility of the stretchable semiconductor film was enhanced up to threefold compared to the neat (unsheared) film and maintained the same up to *ε* = 100%.

### 2.3 Electrospun Nanofiber Interfaces

**Figure 5:**
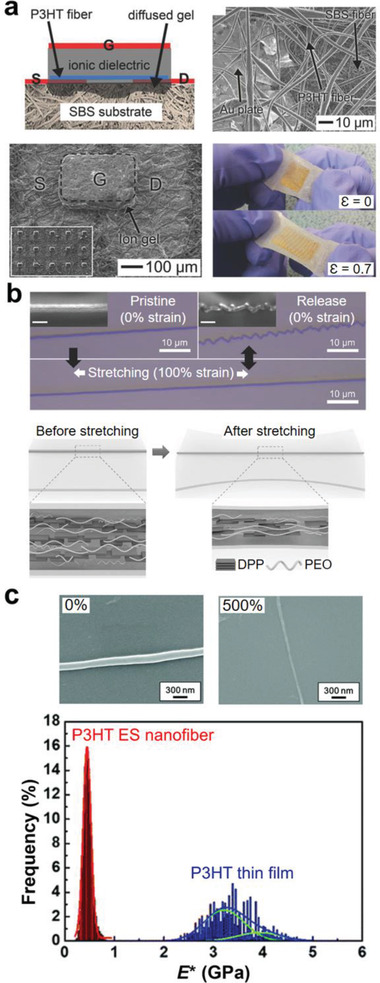
Electrospun nanofiber‐based stretchable polymer semiconductors and substrates. a) Stretchable polymer transistors array constructed with an electrospun semiconducting nanofiber channels, an elastomer nanofiber mat substrate, an ion‐gel dielectric layer, and Au plate‐based stretchable electrodes. Reproduced with permission.^[^ ^82^ ^]^ Copyright 2014, Wiley‐VCH. b) Stretchable semiconductor nanofiber of semiconducting polymer and poly(ethylene oxide) (PEO) composite. PEO acts as a molecular binder to reduce the modulus of the composite nanofibers. Reproduced with permission.^[^ ^83^ ^]^ Copyright 2018, Wiley‐VCH. c) Stretchable P3HT semiconductor nanofiber and the modulus difference between the P3HT nanofibers and the P3HT thin films. Reproduced with permission.^[^ ^84^ ^]^ Copyright 2020, Royal Society of Chemistry.

The use of curved and infinitely long electrospun organic nanofibers is one of the strategies for manufacturing stretchable device components.^[^ ^79^, ^80^, ^81^, ^82^, ^83^, ^84^ ^]^ Organic nanofibers have been widely used as a support matrix for inorganic conductive materials in fabricating stretchable conductors,^[^ ^79^, ^80^, ^81^ ^]^ but studies on the organic–organic interfaces of organic nanofibers have been rarely conducted. High‐molecular‐weight soft polymers such as poly(*ε*‐carprolactone) (PCL) and PEO have been mixed with rigid conjugated polymers to improve the electrospinnability of the relatively low‐molecular‐weight conjugated polymers.^[^ ^82^, ^83^, ^84^ ^]^ Shin et al. developed a stretchable and reliable array of polymer transistors by forming the electrospun P3HT:PCL composite nanofibers on the electrospun polystyrene‐*block*‐polybutadiene‐*block*‐polystyrene (SBS) elastomer nanofiber mat (**Figure** **5**a).^[^ ^82^ ^]^ An ion‐gel polyelectrolyte dielectric layer deposited on the P3HT:PCL nanofibers penetrated both the nanofiber channels and the substrate mats. The interpenetrating network structure was formed in the ion‐gel layer, the porous channel layer, and the substrate. The network provided the transistors with high mechanical stability. The transistor array operated reliably at 70% tensile strain and even after 1500 stretching cycles.

Lee et al. reported the stretchable single organic semiconductor nanofibers composed of fused thiophene diketopyrrolopyrrole (FT4‐DPP) and high‐molecular‐weight PEO (Figure 5b).^[^ ^83^ ^]^ Because PEO has a low glass transition temperature and low modulus (230 MPa), it acts as a molecular binder which increases the mechanical ductility by reducing the modulus of the FT4‐DPP:PEO composite nanofibers. The modulus of pure FT4‐DPP film (960 MPa) was reduced to 303 MPa in FT4‐DPP:PEO nanofibers. Unlike the pure FT4‐DPP nanofibers, the composite nanofibers were fully elongated while maintaining a continuous shape at *ε* = 100%. Under stretching, the charge transport mobility decreased due to the weakened *π*–*π* interactions between the DPP domains; however, the nanofibers were not broken because the PEO binder prevented the crazes from developing further into cracks in the composite nanofibers. After the strain was released, the nanofibers were folded with many wrinkles, but charge carriers could still migrate along the fibers. During additional stretching cycles, the wrinkles in the nanofibers were repeatedly folded and unfolded, allowing the transistor to operate reliably at 100% strain.

Electrospun semiconducting polymer nanofibers with a low glass transition temperature were reported to be stretchable by themselves without the soft polymer binders. Chen et al. reported that the electrospinning process could strengthen the low‐crystalline feature of the P3HT and poly{[*N*,*N*′‐bis(2‐octyldodecyl)naphthalene‐1,4,5,8‐bis(dicarboximide)‐2,6‐diyl]‐*alt*‐5,5′‐(2,2′‐bithiophene)} (N2200) nanofibers, and these electrospun semiconducting nanofibers could be highly stretchable (500%) (Figure 5c).^[^ ^84^ ^]^ The P3HT nanofibers possessed a low modulus of 448 MPa, in contrast to the high modulus (3930 MPa) of the crystalline P3HT films. It has been elucidated that such remarkable stretchability and low modulus are attributed to the low glass transition temperature and the low density of physical crosslinking points (i.e., the crystalline regions) of the P3HT (or N2200) nanofibers. The low density rendered a significant portion of amorphous region to release the external strain.

## 3 Design of Nano/Microscale Organic–Inorganic Interfaces

Currently, the active materials for high device performance are inorganic materials.^[^ ^85^, ^86^, ^87^, ^88^, ^89^, ^90^, ^91^, ^92^, ^93^, ^94^, ^95^, ^96^, ^97^, ^98^, ^99^, ^100^, ^101^, ^102^, ^103^, ^104^, ^105^, ^106^, ^107^, ^108^, ^109^, ^110^, ^111^, ^112^, ^113^, ^114^, ^115^, ^116^, ^117^, ^118^, ^119^, ^120^ ^]^ Because the inorganic materials have a small strain at break (*ε*_b_) (typically, *ε*_b_ < 1%), their composites with elastomeric polymers have been used to obtain stretchability. In the inorganic–organic nanocomposites, homogeneous spatial distribution of the inorganic fillers and strong filler–matrix adhesion is important to secure the mechanical electrical stability under repeated cycles of large strains. Due to the weak inorganic–organic interfaces, delicate control of the interfacial adhesion is required in the inorganic nanocomposites.

Materials in nanoscale have large attractive energy compared to the bulk counterparts due to the large surface‐to‐volume ratio.^[^ ^85^ ^]^ The interparticle attractive potential energy (*ψ*_A_) has been understood using the Hamaker constant (*A*) in the Lifshitz theory with van der Waals energy. *ψ*_A,spheres_ between two spherical nanoparticles (NPs) with equal radius and *ψ*_A,plates_ between two plates of equal thickness are expressed as follows

(1) $$\begin{array}{ccc} \psi_{A,\mathrm{spheres}} & = & -\frac{A}{6}\left[\frac{2R^{2}}{s^{2}+4Rs}+\frac{2R^{2}}{s^{2}+4Rs+4R^{2}}\right.\\ & & \left.+\;\ln\left(\frac{s^{2}+4Rs}{s^{2}+4Rs+4R^{2}}\right)\right] \end{array}$$

(2) $$\psi_{A,\mathrm{plates}}=\;-\frac{A}{12\pi }\left(\frac{1}{s^{2}}+\frac{1}{{\left(s+2\delta \right)}^{2}}-\frac{2}{{\left(s+\delta \right)}^{2}}\right)$$

where *R* is the radius of the particle, *δ* is the thickness of the plates, and *s* is the separation of surfaces along the line of centers.^[^ ^86^ ^]^ When the radius of uniform‐sized spherical particles is much larger than the separation distance (*R* ≫ *s*), which is applicable to highly concentrated composites, *ψ*_A,spheres_ can be simplified, $\psi_{A,\;\mathrm{spheres}}=\;-\frac{AR}{12s}$. Thus, the Hamaker constant is one of the governing parameter determining the attractive potential between nanostructured materials. Due to the large Hamaker constants of inorganic materials (typically, 16.2–45.5 × 10^−20^ J for metals, 10–15.5 × 10^−20^ J for metal oxides) compared to those of polymers (6.15–6.6 × 10^−20^ J),^[^ ^86^ ^]^ aggregation of inorganic nanomaterials takes place readily both in the solution phase of ink and in the polymer matrix of films.

**Figure 6:**
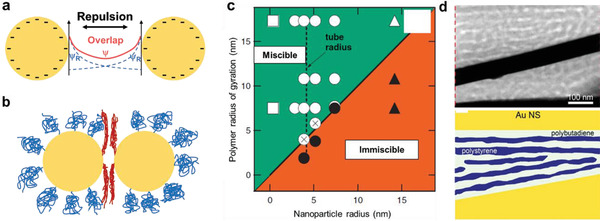
Interface design principles for organic–inorganic nanocomposites. a) Electrostatic stabilization of the particles in a polar solution phase. The electrostatic repulsive potentials (*ψ*_R_) from approaching particles overlap to form an energy barrier and prevent agglomeration of the particles. b) Steric stabilization by long polymers adsorbed at the particle surfaces. The polymer chains are aligned perdicularly to the particle approaching direction. The alignment reduces the entropy of the polymer chain so that the particles can be stable without aggregation. c) Miscible/immisible transition depending on the ratio between the polymer radius of gyration and the particle radius. Particles are immiscible when the radius is larger than the polymer radius of gyration. Reproduced with permission.^[^ ^93^ ^]^ Copyright 2006, AAAS. d) Physical adhesion between Au nanosheets and SBS block copolymer. Polybutadiene soft block selectively adhered to the Au surfaces and formed strong adhesion. Reproduced with permission.^[^ ^96^ ^]^ Copyright 2017, American Chemical Society.

As the fabrication of stretchable devices usually includes a solution process, stable dispersion of the nanostructured inorganic materials in a solution phase is required. The enthalpy‐driven stabilization is based on the Derjaguin–Landau–Verwey–Overbeek (DLVO) theory, and the entropy‐driven stabilization is enabled by the surfactant polymers adsorbed at the nanomaterial surfaces. The DLVO theory suggests that the net electrostatic repulsive potential, *ψ*_R_ = *ψ*_o_ exp(−*κx*), exists between particles through a liquid medium. When separation between two particles is shorter than the reciprocal of electric double‐layer thickness (*κ* ^−1^, the Debye length), agglomeration can be avoided if *ψ*_R_ > *ψ*_A_ because *ψ*_R_ from the particles overlap and sets an energy barrier against interparticle collision (**Figure** **6**a).^[^ ^87^ ^]^ By making the surface of nanomaterials charged with small ionic ligand molecules, this enthalpy‐driven approach has been widely used to produce stable suspension of nanomaterials in polar solvents.^[^ ^88^ ^]^ To prepare stable nanomaterial ink regardless of the polarity of the solvent, attachment of long polymer chains at the nanomaterial surface has been the successful approach.^[^ ^89^ ^]^ When two particles approach each other, the adsorbed polymer chains are aligned perpendicularly to the approaching axis (Figure 6b). This chain alignment greatly reduces the entropy of the polymer chains and increases the free energy of the system, thus the polymer chains push back the approaching particles and retain the initial high‐entropy conformation (steric stabilization). This entropy‐driven stabilization has been used in most hydrophobic nanomaterial ink.^[^ ^90^ ^]^ In hydrophilic dispersions, charged polymers are often used to combine the electrostatic and the steric stabilization (electrosteric stabilization).^[^ ^91^ ^]^

Even when the nanoparticle dispersion in the solution phase is stable, the particles can aggregate during the coating process or the thermal annealing process.^[^ ^92^ ^]^ Unless the composite film is prepared by kinetic quenching through fast solvent evaporation or freeze drying, thermodynamically stable composite is desired. In the nanocomposite films, the inorganic–organic interfacial stability depends on various parameters such as particle species, particle size versus polymer chain dimension, particle shape and concentration, rigidity of the particle, and thermodynamic interaction with the polymer chains. Thermodynamically stable dispersion of the nanoparticles in polymer matrix is enhanced when the radius of gyration of the matrix polymer (usually, in the range of 5–50 nm) is larger than the radius of the particle (Figure 6c).^[^ ^93^ ^]^ The polymer radius of gyration increases with nanoparticle concentration. Addition of particles with relatively bigger particles leads to polymer chain expansion, which is entropically unfavored due to the extended chain conformations, however enthalpically favored due to the increased molecular contact. As most nanoparticles used in conductive nanocomposites are often bigger than the polymer radius of gyration, strong interaction with the polymer matrix is required to maintain the system to be enthalpically favored.^[^ ^90^ ^]^ Formation of covalent bonds between the functionalized surfactants and polymer matrix can create a strong interface.^[^ ^94^ ^]^ Dynamic physical bonds through the hydrogen bonding and Zwitterionic bonding is known to effectively release the stress and increase the stretchability.^[^ ^95^ ^]^ Use of thermoplastic block copolymer matrix can generate stable interfaces because the soft blocks are selectively adhered to the inorganic filler surfaces and they provide good adhesion. Cho et al. revealed that the polybutadiene block made the interface with amine‐functionalized Au nanosheets in the SBS/Au nanosheet composite (Figure 6d).^[^ ^96^ ^]^ The composite maintained strong interfacial adhesion so that pulling out the Au nanosheets with adhesive tape was not possible and phase separation of the Au nanosheets did not occur by thermal annealing.

The process of manufacturing the composite films may lead to difference in the physical properties. The solution casting is advantageous in preparing uniformly distributed composite films.^[^ ^97^ ^]^ However, since the polymer chains are solidified as the solvent evaporates, the polymer chains near the surface are more stretched, thus mechanical stress is generated in the thickness direction. This residual stress formed during the casting process can be removed by thermal annealing or adding plasticizer in the polymer matrix.^[^ ^92^ ^]^ The casting process often generates a gradient distribution of the nanomaterials, which can be a problem in obtaining reliable electrical mechanical properties. The large polymer coverage at the top surface caused by the relatively small surface energy of the polymer matrix is not readily controllable.^[^ ^98^ ^]^ Strong interfacial adhesion is critical in stretchable electronics. Applying tensile stress at the weakly bonded organic–inorganic interface creates voids and delamination due to concentrated stress.^[^ ^99^ ^]^ Once strong interfacial adhesion is assured, nanomaterials with a large aspect ratio (length/diameter for nanowires, width/thickness for nanoflakes) are desired for reinforcement of the polymer matrix, however not desirable for high stretchability. It is because the energy transfer from the matrix to the nanomaterials takes place mainly in the central part of the fillers, and the energy transfer near the edges of the fillers is not considerable.^[^ ^98^ ^]^ On the other aspect, the nanomaterials with a high aspect ratio are advantageous for electrical percolation. Therefore, design of a composite is up to the target property to be achieved. Generally, the composites with high stretchability and good electrical conductivity can be prepared using a small amount of long 1D fillers or using a large amount of 2D flakes or nanoparticles.^[^ ^100^ ^]^

In general, there have two types to control organic–inorganic interface: surface functionalization^[^ ^101^, ^102^, ^103^, ^104^, ^105^, ^106^, ^107^, ^108^, ^109^, ^110^ ^]^ and addition of additives.^[^ ^109^, ^110^, ^111^, ^112^, ^113^ ^]^ Surface functionalization of inorganic materials with organic molecules has been well‐developed in the solution‐based synthesis of nanostructured inorganic materials, including thiol chemistry on metal surfaces,^[^ ^101^ ^]^ silane chemistry on oxide surfaces,^[^ ^102^ ^]^ creating functional groups on metals and oxides by plasma exposure and ozone treatment,^[^ ^103^, ^104^ ^]^ radical formation and subsequent surface adsorption,^[^ ^105^, ^106^, ^107^ ^]^ coating with charged small molecules,^[^ ^108^ ^]^ surface wrapping or adsorption by long polymer chains,^[^ ^114^ ^]^ etc. Due to the poor miscibility of the nanomaterials with the polymer matrix or the polymer solution, design of the functional groups on the nanomaterial surface has been focused on increasing the compatibility with the polymer. Introducing additives such as surfactants and lubricants can be a simple way to increase the compatibility of the nanomaterials with the polymer matrix. Although the additives do not have chemical bonds with the inorganic surfaces, they are located at the interface and decrease the interfacial energy so that the mechanical stress can be transferred readily through the interface.

### 3.1 Liquid Metal–Nanoparticle Interface

**Figure 7:**
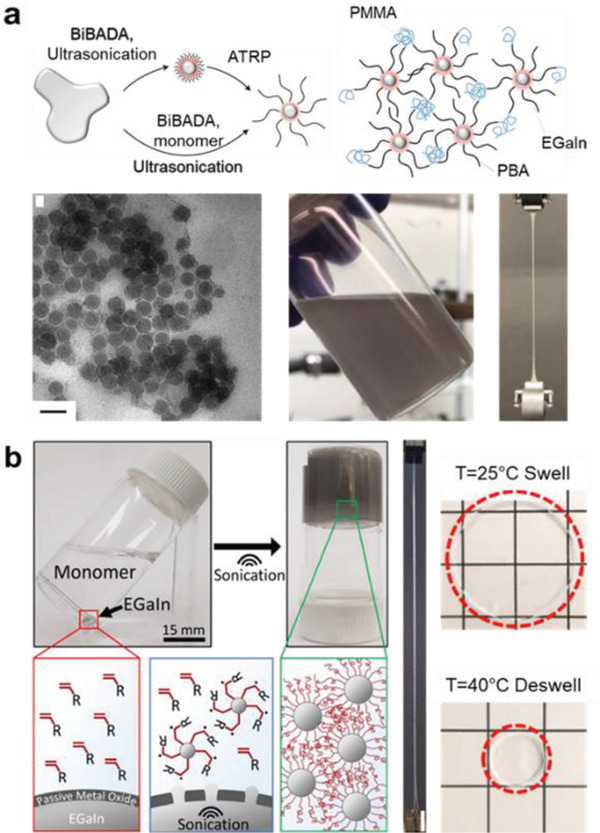
Organic functionalization of liquid metal NPs. a) EGaIn oxide functionalization with stretchable block copolymers by using the ATRP synthesis. The film prepared by casting the NP ink showed excellent stretchability. Reproduced with permission.^[^ ^105^ ^]^ Copyright 2019, Springer Nature. b) Free radical polymerization performed directly on bare liquid metal surfaces. The composite hydrogel reversibly responded during swelling/deswelling upon cooling/heating around 33 °C. Reproduced with permission.^[^ ^106^ ^]^ Copyright 2019, American Chemical Society.

Recently, liquid metals that are eutectic metal alloys with a melting point below the room temperature have attracted intensive attention as stretchable circuit lines.^[^ ^121^, ^122^, ^123^, ^124^ ^]^ The shortcomings of the liquid metals are poor wettability on most substrates, continuous alloy formation with other metals (for instance, the metal pads (Au, Ag, Cu) on the operating chip), and ironically the good fluidity which is not compatible with conventional device manufacturing lines. One possible approach is synthesizing liquid metal NPs functionalized with organic molecules and producing composites with elastic polymers. This aim has not been achieved yet, but there have been some advances made recently. Yan et al. functionalized the oxide surface of the eutectic indium gallium alloy (EGaIn) NPs with various polymers including poly(methyl methacrylate) (PMMA), poly(*n*‐butyl acrylate) (PBMA), poly(2‐dimethyl amino)ethyl methacrylate) (PDMAEMA), and poly(*n*‐butyl acrylate‐*block*‐methyl methacrylate) (PBA‐*b*‐PMMA) (**Figure** **7**a).^[^ ^105^ ^]^ They used the surface‐initiated atom transfer radical polymerization (ATRP) by applying ultrasonication for bulk EGaIn in the presence of monomer and 12‐(2‐bromoisobutyramido)dodecanoic acid (BiBADA). The surface of the EGaIn NPs was functionalized with BiBADA and polymerization was initiated from the end of BiBADA through the ATRP process. The functionalized EGaIn NPs were uniform sized and well‐dispersed in organic solvents. The printed stretchable thermoplastic polymer composite film could be stretched over *ε* = 700% strain. Such high stretchability was attributed to the viscoelastic characteristics of the liquid metal NPs. Ma et al. functionalized the EGaIn NPs with polyacrylamide (PAAm) and poly(N‐isopropylacrylamide) (PNIPAAm) (Figure 7b).^[^ ^106^ ^]^ They initiated polymerization by ultrasonication in an aqueous monomer solution without initiator. The free radical polymerization formed high molecular weight polymers grafted to the NP surface and simultaneously produced a hydrogel with an extreme stretchability (*ε* ≈ 1500%). The PNIPAAm hydrogel showed reversible response of swelling/deswelling upon cooling/heating around 33 °C. Ren et al. functionalized the EGaIn NPs with thiol to fabricate superconducting NPs by sonicating the bulk EGaIn in an ethanol solution of ethyl 3‐mercaptopropionate.^[^ ^101^ ^]^ The NPs with thiol self‐assembly monolayer were dispersed in various solvents such as ethanol, acetone, and water. The NPs retained the superconducting property shown in bulk EGaIn and had excellent wettability to most material surfaces (metals, metal oxides, polymers).

### 3.2 Nanomaterial Interfaces for Tough Hydrogels

**Figure 8:**
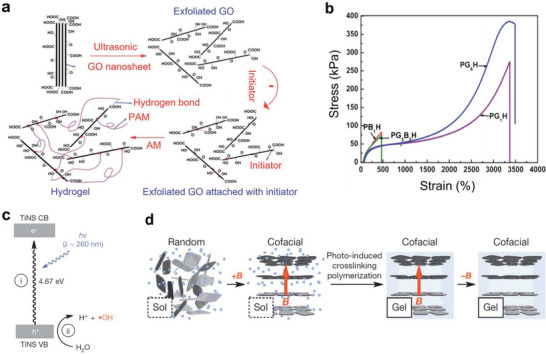
Hydrogel composites with controlled mechanical properties by using inorganic nanomaterials. a) Fabrication process of tough and highly stretchable GO/PAAM composite hydrogel. b) Stress–strain curves of the GO‐based hydrogels and conventional chemically crosslinked hydrogels. Reproduced with permission.^[^ ^107^ ^]^ Copyright 2012, Royal Society of Chemistry. c) Hydroxyl radicals produced by UV irradiation on Ti NS. d) Scheme of the quasi‐crystalline layer of Ti NS formed by external magnetic field. Photo‐induced crosslinking inhibited the relaxation of the anisotropically arranged Ti NSs after removing the magnetic field. Reproduced with permission.^[^ ^108^ ^]^ Copyright 2015, Springer Nature.

Hydrogel has recently attained increasing interest as a stretchable medium due to the excellent biocompatibility requested in the skin‐mounted and implanted devices.^[^ ^107^, ^108^, ^117^, ^118^, ^125^ ^]^ Conventional hydrogels are mechanically weak due to the irregular distribution of the crosslinking points. Liu et al. fabricated a tough and highly stretchable graphene oxide (GO)/PAAm composite hydrogel (**Figure** **8**a).^[^ ^107^ ^]^ GO has strong interfacial interaction with the polymer matrix due to the functional groups (hydroxyl, epoxide, carboxyl acid, etc.) on its surface. The functional groups made the GO dispersed homogeneously and improved the mechanical properties of the composite. GO was exfoliated by ultrasonication, and an initiator (potassium peroxydisulfate) was attached on the GO nanosheet surfaces. The free radical polymerization of acrylamide propagated from the initiator to produce the polymers bridging the GO nanosheets. The mechanical properties of the GO nanosheet‐based hydrogels and the conventional hydrogels crosslinked with *N,N*′‐methyl‐enebisacrylamide (BIS) are compared in Figure 8b. In the figure, PG*_m_*H refers to GO nanosheets used as crosslinker and PB*_m_*H indicates BIS. The PG*_m_*B*_m_*H refers to GO made with the assistance of BIS co‐crosslinkers (*m* stands for 10 × GO (or BIS)/water (g L^−1^)). Tensile strength of PGH was four to five times higher than that of PBH, and the toughness was more than ten times higher. However, PG_3_B_3_H had poor tensile properties compared to PG_6_H due to their high crosslinking density. PG_6_H exhibited the maximum tensile strength (385.0 kPa), elongation at break (*ε* = 3435%), and toughness (4.74 MJ m^−3^).

Similar approaches were suggested to enhance the mechanical property of the nanocomposite hydrogel (NC gel) obtained through free radical polymerization. Harahuchi et al. prepared PNIPAm‐based water‐swellable nanoclay gel.^[^ ^117^ ^]^ They found that the average distance between the exfoliated nanoclays was equivalent to the crosslinking distance. Wang et al. used PNIPAm and Au NPs to fabricate a composite gel with controlled swelling degree and thermal phase transition.^[^ ^118^ ^]^ Liu et al. produced a composite hydrogel having anisotropic mechanical properties dominated by electrostatic repulsion between negatively charged unilamellar titanate nanosheets (Ti NSs) (Figure 8c).^[^ ^108^ ^]^ Irradiation with ultraviolet light (wavelength (*λ*) < 260 nm) to the semiconducting Ti NSs in the presence of N,N‐dimethylacrylamide produced hydroxyl radicals which triggered the in situ radical polymerization of acrylamide. The high density negative surface charges ensured stable dispersion of the Ti NSs without surfactants in aqueous media. When a magnetic field (over 10 T) was applied, the randomly oriented Ti NSs formed a layered structure with a quasi‐crystalline order on a macroscopic length scale (Figure 8d). Relaxation of the anisotropically arranged Ti NSs was completely inhibited by hydrogelation. This Ti NSs hydrogel composite enabled excellent directional vibration, which was possible due to the enhanced friction in the vertical direction of the NSs and the reduced friction in the parallel direction.

### 3.3 Metal Interfaces for Conductive Stretchable Composites

Most nanocomposites investigated for the stretchable devices so far have been the conductors, used either as strain‐insensitive electrodes or as strain‐responsive sensors.^[^ ^126^, ^127^, ^128^ ^]^ Selecting the appropriate filler is one of the key factors for successful nanocomposites with high conductivity and stretchability. Electrical percolation threshold strongly depends on the size and shape of the fillers, which is larger in the order of 1D (wire), 2D (plate), and 0D (particle). Ag nanowires (Ag NWs) with a high aspect ratio reduced the percolation threshold so that highly transparent stretchable electrodes could be readily obtained.^[^ ^129^, ^130^, ^131^, ^132^, ^133^, ^134^, ^135^, ^136^, ^137^ ^]^ Ag flakes have been widely used for stretchable composite electrodes because the Ag flake inks could be immediately adopted in the conventional 2D and 3D‐printed electronics.^[^ ^102^, ^111^, ^112^, ^119^, ^120^, ^139^ ^]^ In this section, we deal with the recent achievements of the stretchable conductive composites using Ag NWs and Ag flakes, focusing on the interfacial design aspects.

**Figure 9:**
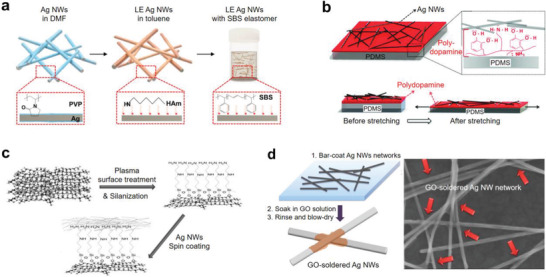
Stretchable Ag NW composites through interface engineering. a) Ligand exchange (LE) of the Ag NWs to assure homogeneous Ag NWs dispersion and strong interface with the elastomer matrix. Reproduced with permission.^[^ ^131^ ^]^ Copyright 2015, American Chemical Society. b) Stretchable Ag NWs electrode having strong Ag NW–substrate interface by modifying the PDMS surface with polydopamine. Reproduced with permission.^[^ ^132^ ^]^ Copyright 2012, American Chemical Society. c) Surface treatment of the PDMS substrate by self‐assembled molecules with different functional groups to enhance the Ag NW–substrate interfacial adhesion. Reproduced with permission.^[^ ^133^ ^]^ Copyright 2014, Wiley‐VCH. d) Physical soldering of the Ag NW junctions with graphene oxide (GO) nanosheets. The soldered Ag NW network showed enhanced mechanical electrical stretchability. Reproduced with permission.^[^ ^137^ ^]^ Copyright 2014, American Chemical Society.

Strong interface between Ag NWs and the surrounding organic/polymer matrix can give stable and large stretchability. Ag NWs have been synthesized in the presence of polymeric surfactant, usually poly(vinylpyrrolidone (PVP), because of the selective adsorption to the (100) surface of the Ag NWs.^[^ ^130^ ^]^ However, the polar PVP does not have good compatibility with the thermoplastic polymer matrix such as SBS and SEBS. Choi et al. demonstrated that ligand exchange of the Ag NWs could allow homogeneous dispersion in the elastomer matrix (**Figure** **9**a).^[^ ^131^ ^]^ They exchanged the PVP surfactant by hexylamine (HAm) ligand for homogeneous dispersion of the Ag NWs in a nonpolar solution of SBS. The printed electrode had good spatial homogeneity of the Ag NWs in the polymer matrix with a strong interface, which brought not only the uniform mechanical and electrical properties but also the high processability and patternability. Adhesion of Ag NWs to the elastomer substrate is another important issue for obtaining reliable stretchable electrodes because weak adhesion increases the resistance of the electrode and causes mechanical failure during repeated stretching cycles. Physical embedding of Ag NWs in the elastomer substrate and enhancing the chemical interaction at the interface have been proposed to solve the issue.^[^ ^100^, ^129^ ^]^ Akter et al. fabricated a stretchable and transparent Ag NWs electrode by modifying the PDMS substrate with polydopamine (Figure 9b).^[^ ^132^ ^]^ A strong binding between the dopamine and the Ag NWs facilitated uniform spray‐deposition of Ag NWs and allowed strong adhesion of Ag NWs to the substrate. Molecular modification of the elastomer substrate was also investigated to enhance the adhesion. Lee et al. investigated the adhesion of Ag NWs to the substrates modified by five different functional head groups, including NNH_2_—, NH_2_—, SH—, Cl—, and O‐silane derivatives (Figure 9c).^[^ ^133^ ^]^ They silanized the surface of the oxygen plasma‐treated PDMS and self‐assembled the functional groups. They found that the amine (NNH_2_—) functional head groups could form the strongest adhesion to the Ag NWs due to the highest sigma (*σ*)‐donating polarity. The Si(NNH_2_)‐treated PDMS substrate showed the largest conductivity (sheet resistance of 27 Ω sq^−1^), stretchability (*ε* = 30%), and reasonable durability under 1000 stretching cycles.

The inter‐nanowire junction is also an important issue to assure high electrical conductivity and mechanical durability of the stretchable Ag NWs electrodes. Conventional soldering processes such as high‐temperature annealing^[^ ^134^ ^]^ and flash light sintering^[^ ^135^, ^141^ ^]^ have been widely investigated. Recently, the interfacial soldering between Ag NWs has been attempted to increase the mechanical strength of the inter‐nanowire junctions.^[^ ^136^, ^137^ ^]^ Chen et al. soldered the Ag NWs by preferentially generating Ag NPs over the NW junctions by chemical reduction of Ag precursors.^[^ ^136^ ^]^ The soldered Ag NWs reinforced the conducting network and exhibited stable electrical conduction at a large uniaxial strain (*ε* = 120%). Without chemical reaction, Liang et al. soldered Ag NW junctions by wrapping around the junctions with GO nanosheets (Figure 9d).^[^ ^137^ ^]^ The GO nanosheets adhered to the Ag NWs and soldered the junctions because a large number of oxygen functional groups on the GO facilitated strong binding to Ag NWs. Resultantly, high conductivity was obtained from the electrode (sheet resistance of 26 Ω sq^−1^). In addition, the high Young's modulus (≈0.25 TPa) of the GO sheets could make strong and tough bonding of the Ag NWs under stretching.

**Figure 10:**
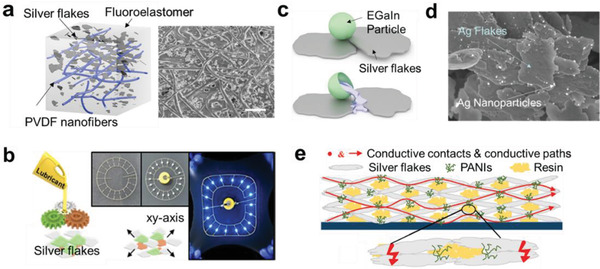
Stretchable Ag flake nanocomposites with interface engineering. a) Ag flake and fluoroelastomer composite embedded in electrospun PVDF nanofibers. Reproduced with permission.^[^ ^114^ ^]^ Copyright 2019, American Chemical Society. b) Silver flake composite with silicon oil lubricant for uniform stress distribution. Reproduced with permission.^[^ ^110^ ^]^ Copyright 2019, American Chemical Society. c) Silver flake composite with EGaIn particles added to improve the electrical contact between the silver flakes. Reproduced with permission.^[^ ^111^ ^]^ Copyright 2018, Wiley‐VCH. d) Fabrication of Ag flakes decorated with Ag NPs formed by adding KI surfactant. Reproduced with permission.^[^ ^112^ ^]^ Copyright 2019, American Chemical Society. e) Ag flake and conducting polymer (PANI) composite electrode. PANI improved electrical connection between the Ag flakes. Reproduced with permission.^[^ ^113^ ^]^ Copyright 2018, Royal Society of Chemistry.

Various interfacial designs to increase the stretchability of the Ag flake composites have been reported.^[^ ^110^, ^111^, ^112^, ^113^, ^114^, ^119^, ^120^ ^]^ Jin et al. fabricated Ag flake composite embedding electrospun poly(vinylidene fluoride) (PVDF) nanofibers (**Figure** **10**a).^[^ ^114^ ^]^ The pores with average size of 2.9 µm among the nanofibers were filled with the Ag‐rich conductive composite, resulting in high electrical conductivity of 9900 S cm^−1^. The PVDF nanofiber improved the mechanical toughness (6.58 MJ m^−3^) so that the composite could be stretched up to *ε* = 800% with the growth of cracks. Hwang et al. added silicon rubber oil to the Ag flake composite as a lubricant to reduce the friction between the Ag flakes (Figure 10b).^[^ ^110^ ^]^ The distribution of elastic modulus across the entire composite film is often uneven when the conductive particles are not uniform in size. This inhomogeneous modulus leads to localized stress concentration and causes mechanical fracture under a small tensile strain. Finite element method study showed that addition of the lubricant (22 vol%) improved the dispersion uniformity of the Ag flakes, thus also the stress distribution while being stretched. They fabricated a circular circuit using the nozzle printing and connected a battery and light‐emitting diodes (LEDs). They reported stable LED operation with a 260 µm printed line under biaxial stretching.

One of the challenges in the conductive composites is a trade‐off between the modulus and conductivity. Increasing the metal fraction enhances the conductivity but reduces the stretchability due to the increased modulus. Addition of a small amount of soft conductive materials has been proposed as an effective way to achieve both the high conductivity and stretchability. Wang et al. introduced EGaIn microparticles (MPs) in an Ag flake composite (Figure 10c).^[^ ^111^ ^]^ The EGaIn MPs were mixed with Ag flakes and poly(ethylene vinyl acetate) (PEVA). The EGaIn MPs were cracked under stretching and bridged the Ag flakes. Adding 40 wt% of EGaIn MPs brought a high conductivity (8331 S cm^−1^) without considerable degradation even at *ε* = 1000%. Guo et al. effectively introduced Ag NPs between the Ag flakes (Figure 10d).^[^ ^112^ ^]^ They found that there were some remaining surfactants on the commercial Ag flakes and iodized the surfactants by using potassium iodide (KI). When the the KI‐treated Ag flakes were exposed to sunlight for 1 h, the surface silver oxide of the Ag flakes were converted into Ag NPs. The Ag NP‐decorated Ag flake composite showed an improved electrical conductivity (1.1 × 10^4^ S cm^−1^) compared to the bare Ag flake composite (1.42 × 10^−6^ S cm^−1^) and showed stable resistance up to *ε* = 119%. Wen et al. improved electrical interface of Ag flakes by adding a conducting polymer (polyaniline, PANI) in between the Ag flakes (Figure 10e).^[^ ^113^ ^]^ PANI provided a coherent conduction pathway between the Ag flakes, hence a small amount of addition (0.5 wt%) increased the electrical conductivity from 7.98 × 10^2^ to 2.7 × 10^3^ S cm^−1^. Matsuhisa et al. used selective phase separation of an Ag flake layer which were localized at the surface of a composite.^[^ ^120^ ^]^ Surface accumulation of the Ag flakes was induced partially by self‐assembly of 4‐methyl‐2‐pentanone and the reduced surface energy of the Ag flake by the adsorbed fluorine surfactant. The surface accumulation took place after annealing the printed composite at 80 °C. Although this approach did not result in high conductivity (738 S cm^−1^ at *ε* = 0%), it improved the stretchability up to *ε* = 215%.

### 3.4 Interlayer Interface to Minimize the Strain Effect

**Figure 11:**
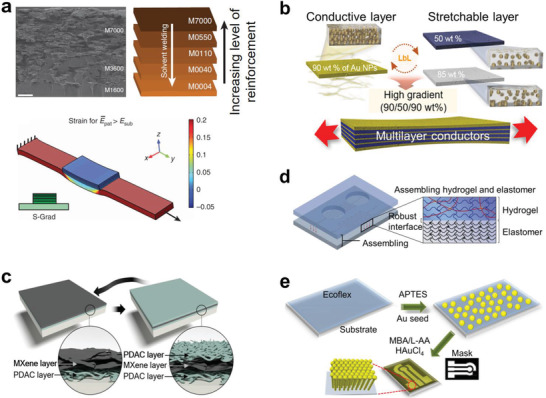
Multilayered composites. a) Vertically stacked layers with gradient modulus to suppress delamination of hard segments on soft substrates. Reproduced with permission.^[^ ^115^ ^]^ Copyright 2012, Springer Nature. b) Alternating multilayer stacking of high‐ and low‐modulus layers to obtain high stretchability and metallic electrical conductivity. Reproduced with permission.^[^ ^138^ ^]^ Copyright 2019, AAAS. c) 2D metal carbides and positively charged polyelectrolyte layer‐by‐layer composite. Reproduced with permission.^[^ ^116^ ^]^ Copyright 2018, AAAS. d) Hydrogel‐elastomer hybrid substrate with strong interfacial toughness. Reproduced with permission.^[^ ^140^ ^]^ Copyright 2016, Springer Nature. e) APTES surface treatment of elastomer surface to attain stable interface with Au metal seeds, followed by growth of Au nanowires on the patterned area only. Reproduced with permission.^[^ ^102^ ^]^ Copyright 2018, American Chemical Society.

Multilayers of the composites with modulus gradient improved the stretchability and conductivity.^[^ ^115^, ^116^, ^138^ ^]^ To overcome the stress concentration by the large modulus difference, Libanori et al. stacked multiple layers of different modulus films to provide a modulus gradient with the highest modulus layer on the top surface (**Figure** **11**a).^[^ ^115^ ^]^ Elastic moduli of these multilayers were controlled by adjusting the amount of polyurethane, inorganic nanoplatelets (laponite), and inorganic microplatelets (alumina). The gradient distribution of the stress suppressed delamination of the hard segments on the soft substrate, thus the multilayer film had a stretchability up to *ε* = 350%. Gu et al. demonstrated a multilayer electrode which is biaxially stretchable up to *ε* = 300% by stacking the Au NP/polyurethane composites with a gradient concentration of Au NPs (Figure 11b).^[^ ^138^ ^]^ High modulus conductive layer and low‐modulus stretchable layer were coated by repeating the alternate filter process. The stretchable low modulus layer dissipated the applied stress while being stretched. This multiple layer interface resulted in metallic conductivity (1.57 × 10^4^ S m^−1^). An et al. showed a multilayer composite of 2D conductive materials.^[^ ^116^ ^]^ The negatively charged 2D metal carbides (MXenes) were assembled with positively charged polyelectrolytes through the layer‐by‐layer (LbL) process and then coated on a stretchable polymer substrate (Figure 11c). This conformal MXene multilayer maintained its conductivity of 20 S cm^−1^ under mechanical deformations.

Hydrogel‐based composites have been investigated both as stretchable substrates and strain sensors.^[^ ^107^, ^117^, ^118^ ^]^ To attain fast elastic response, the hydrogel substrates are often used as a double layer with a hydrophobic polymer elastomer. Due to the big difference of hydrophilicity, the hydrogels and the polymer elastomers typically have poor adhesion. Yuk et al. reported hydrogel‐elastomer hybrid substrate with a strong interface of interfacial toughness over 1000 J m^−2^ (Figure 11d).^[^ ^140^ ^]^ They modified the cured elastomer surface with benzophenone and activated grafting of the hydrogel polymer chains. In this way, an elastomer layer and a hydrogel layer prepared separately could be adhered strongly. Self‐assembled monolayers (SAMs) can be an effective versatile strategy for securing strong interfacial adhesion. Zhai et al. demonstrated Enokitake mush room‐like gold NWs which were grown directly on elastomeric substrates and used them as stretchable electrodes (Figure 11e).^[^ ^102^ ^]^ They pretreated the Ecoflex substrate with O_2_ plasma and immersed it in an ethanol solution containing (3‐aminopropyl)triethoxysilane (APTES) and Au NPs stabilized by citrate. The vertically aligned Enokitake‐like Au NWs (v‐Au NWs) were grown on the Au seed‐decorated Ecoflex substrate by immersing it in a mixture solution containing HAuCl_4_, 4‐mercaptobenzoic acid (MBA), and l‐ascorbic acid for 3 min. Diameter of the NWs was about 7.8 ± 1.7 nm, and the height could be controlled from 1.5 to 14 µm by adjusting the reaction time. Due to the excellent adhesion to the substrate, the v‐Au NW electrode was not damaged by multiple times of the Scotch tape peel test, and it was stretched up to *ε* = 900%. Lee et al. applied the aminosilane chemistry on the PDMS surface to adhere Ag NWs. After coating the Ag NWs on the amine‐functionalized surface, they used xenon flash light (400–1000 nm, 4 J cm^−2^) to create nanowelded Ag NW network to reduce the contact resistance between the Ag NW interface.^[^ ^141^ ^]^ The Ag NW electrode could be stretched up to *ε* = 50%.

## 4 Design of Mesoscale Interfaces

Electronic devices contain a stack of multiple layers of different materials and often include microsized patterns and domains which have interfaces with other materials. The interface control has been one of the main themes in stretchable electronics because the material properties near the interface determine the stretchability and reliability of the device. Mesoscale interfaces have been designed mainly in three approaches: strain engineering, enhancing the interfacial adhesion, and stacking multiple layers. Strain engineering was applied to the interfaces including the conventional rigid electronic components. Introducing buckling, islands, or high relief structures into the interfaces could adjust the strain applied to the two different layers.^[^ ^142^, ^143^, ^144^, ^145^, ^146^, ^147^, ^148^, ^149^, ^150^, ^151^, ^152^, ^153^, ^154^, ^155^, ^156^, ^157^, ^158^, ^159^, ^160^, ^161^ ^]^ The adhesion between the two layers was increased by introducing microstructured surfaces at the interfaces,^[^ ^142^, ^143^, ^144^, ^145^, ^146^, ^147^, ^148^, ^149^, ^150^, ^151^, ^152^, ^153^, ^154^, ^155^, ^156^, ^157^, ^158^, ^159^, ^160^, ^161^, ^162^, ^163^, ^164^, ^165^, ^166^ ^]^ such as interlocking metal nanopiles,^[^ ^162^ ^]^ hydrogels,^[^ ^164^ ^]^ and SBS block copolymers.^[^ ^165^, ^166^ ^]^ Interfacial adhesion between the multiple device components is an important issue for integrated stretchable devices. In fabricating stretchable devices, the interfacial adhesion between the device layers was strengthened by forming every device component to have similar mechanical and chemical properties,^[^ ^18^, ^167^ ^]^ or weakened for easy delamination by adjusting the adhesion between the two films.^[^ ^170^ ^]^

### 4.1 Strain Engineering in Double‐Layer Interfaces

**Figure 12:**
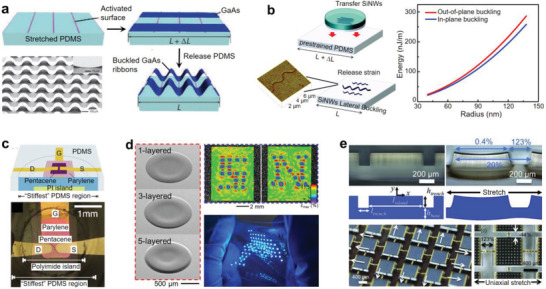
Strain engineering for rigid device components. a) Activation of the adhesion sites on the prestrained PDMS substrate to generate 3D configurations in the buckled GaAs nanoribbons. Reproduced with permission.^[^ ^147^ ^]^ Copyright 2006, Springer Nature. b) In‐plane buckling of the Si NWs and its energy associated with the out‐of‐plane and in‐plane buckling. Reproduced with permission.^[^ ^150^ ^]^ Copyright 2009, American Chemical Society. c) Fabrication of rigid thin‐film transistor on a stiff PDMS region in which a polyimide island was embedded. Reproduced with permission.^[^ ^154^ ^]^ Copyright 2011, American Institute of Physics. d) Stretchable display platform based on inkjet‐printed rigid islands. Reproduced with permission.^[^ ^156^ ^]^ Copyright 2017, Springer Nature. e) Elastomer substrate with high‐relief structures that confined strains at the locations of the interconnections. Reproduced with permission.^[^ ^160^ ^]^ Copyright 2011, Wiley‐VCH.

After Rogers and co‐workers demonstrated the buckling‐based stretchable electronic devices,^[^ ^142^, ^143^, ^144^, ^145^, ^146^, ^147^ ^]^ buckling has been used extensively to fabricate stretchable devices. Buckling occurs when compressive force is exerted to a double layer with a large modulus difference, and the buckled structure depends on the moduli of the interfacing layers, dimension of the buckled film, the prestrain applied to the substrate, the thickness of the substrate, and the interfacial adhesion. Those variables result in different buckling deformations, such as in‐plane buckling, out‐of‐plane buckling, locally delaminated buckling, or a combination of all.^[^ ^142^, ^143^, ^144^, ^145^ ^]^ Initially, the approach was used for changing the Si‐based conventional devices stretchable.^[^ ^146^, ^147^, ^150^ ^]^ Khang et al. produced a stretchable form of Si ribbons structured into out‐of‐plane wavelike geometries.^[^ ^146^ ^]^ Amplitude and wavelength of the wavy Si ribbons could be controlled by the Si thickness and the prestrain applied to the substrate. Sun et al. demonstrated that activating the adhesion sites on the prestrained PDMS substrate by lithographical process can be used in creating 3D configurations in the buckled semiconductor (GaAs and Si) nanoribbons (**Figure** **12**a).^[^ ^147^ ^]^ Changing the prestrain of the substrate and the spacing of the activated stripes could change the shape of the nanoribbons. The buckled semiconductor nanoribbons showed high levels of stretchability (*ε* = 100%) and compressibility (*ε* = −25%). Kim et al. integrated an array of Si nanoribbons on the mechanically neutral plane in a multilayer film composed of ultrathin polymer films and elastomer substrates.^[^ ^6^ ^]^ Combination of the neutral plane and the out‐of‐buckling structure enabled the intrinsically brittle Si materials to be stretchable. Someya and co‐workers adopted printing processes and further developed the out‐of‐plane buckling strategy to fabricate ultralight stretchable devices.^[^ ^148^, ^149^ ^]^ The organic transistors^[^ ^148^ ^]^ and polymer light‐emitting diodes (PLEDs)^[^ ^149^ ^]^ fabricated on the 1.4 µm‐thick polymer substrates attained high flexibility and were stable under extreme bending (bending radius of less than 5 µm) and harsh crumpling. Once the crumpled ultrathin transistors and PLEDs were prepared by applying the prestrain, they were operated stably up to near the applied prestrain.

The buckled form depends on the dimension of the buckled objects. Ryu et al. demonstrated wavy Si NWs by applying the prestrain approach.^[^ ^150^ ^]^ Unlike the out‐of‐buckling Si nanoribbon, the buckling of the Si NWs occurred in the in‐plane direction of the substrate. Theoretical modeling and experimental result indicated that the energy associated with the in‐plane buckling of Si NWs is lower than the out‐of‐plane buckling (Figure 12b). For example, a Si NW of 50 nm radius has energies of 33.9 and 37.6 nJ m^−1^ for in‐plane and out‐of‐plane buckling, respectively. The energy difference of 3.7 nJ m^−1^ is significant for the 1D Si NW as it corresponds to 74 mJ m^−2^ (3.7 nJ m^−1^ divided by the diameter 50 nm) in 2D, which is almost 50% larger than the adhesion energy between Si and PDMS (50.6 mJ m^−2^). The wavelength and amplitude of the buckling changed according to the radius and Young's modulus of the Si NWs. Therefore, from this study, measurements of the dimensions and buckling geometries of the various NWs enabled to obtain Young's modulus of individual wires.

When the buckled form is not preferred in an integrated stretchable device, the “rigid islands” strategy which localizes high modulus regions in an elastic substrate has been used as an alternative way of fabricating stretchable devices. The rigid islands structures with a repetitive stiff‐soft‐stiff system were developed through the modulus engineering. By sequentially arranging the high modulus region and the low modulus region through selective crosslinking or composite formation, the strain could be concentrated only in the desired regions.^[^ ^151^, ^152^ ^]^ The “rigid islands” structure makes possible to integrate the conventional rigid electronic components, free from the strain. Especially, the development of stretchable printed interconnects made possible the active electronic elements located on rigid polymer islands to be successfully incorporated in stretchable devices.^[^ ^153^ ^]^ Graz et al. applied the gradient modulus near the island interface.^[^ ^154^ ^]^ They adjusted photo‐crosslinking to make the overall PDMS substrate soft but the PDMS around the island interface stiff (Figure 12c). They additionally embedded the polyimide (PI) islands inside the stiffest PDMS region to further strengthen the elastomer and fabricated rigid thin‐film transistors directly on the elastomer. The transistors maintained the performance without degradation up to *ε* = 13%. Jung et al. fabricated organic thin‐film transistors (OTFTs) on a polyimide rigid islands and mounted them on the elastomer substrate.^[^ ^155^ ^]^ The polyimide islands on the elastomer surface experienced relatively small strains, thus the OTFTs were mechanically endurable without performance degradation up to *ε* = 50%. Byun et al. presented a stretchable display platform based on inkjet‐printed rigid islands (PRIs) (Figure 12d).^[^ ^156^, ^157^, ^158^ ^]^ Inkjet printing of a polymer solution on the elastomer substrate enabled the formation of the disk‐shaped multilayered PRIs of tunable thickness; and the strain on those PRIs were controlled in a customized way by adjusting the number and shape of the PRI layers, and also the molecular weight of the printed polymers. Kim et al. reported an origami substrate which composed of rigid support fixtures and stretchable interconnects.^[^ ^159^ ^]^ The origami hybrid substrates with rigid epoxy frames and elastomeric joints could be repetitively folded and stretched with an aid of the stretchable interconnects.

The rigid islands structures were made not only by the high modulus polymer islands but also through the structural design of the elastomer substrates. Lee et al. exploited elastomer substrates with high‐relief structures that confined strains at the locations of the interconnections (Figure 12e).^[^ ^160^ ^]^ They designed a PDMS substrate with regularly spaced protruding rectangular islands separated by recessed trenches. Upon *ε* = 20% in the substrate, the strain was concentrated in the trench (*ε* = 123%), however the strain at the islands was negligible (*ε* = 0.4%). They transferred GaAs solar cell modules on the PDMS islands and demonstrated stable operation under biaxial stretching at *ε* = 20%. Cantarella et al. tested various shapes (square, hexagonal, and circular) of the high‐relief islands to improve the stretchability.^[^ ^161^ ^]^ They found that circular pillars have less concentration of the strain than the square and hexagonal pillars.

### 4.2 Strong Interfacial Adhesion in Microstructured Surface

**Figure 13:**
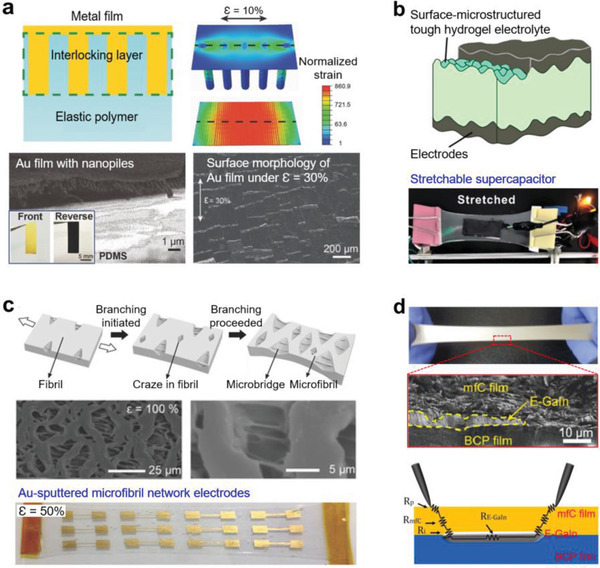
Enhanced interface adhesion with microstructured surfaces. a) Strong adhesion and stretcahbility of a Au film electrode achieved by nanopile interlocking structure at the interface between the Au layer and the elastic substrate. Reproduced with permission.^[^ ^162^ ^]^ Copyright 2017, Wiley‐VCH. b) Microstructured interface for the robust electrode/electrolyte interface in the stretchable supercapacitor. Reproduced with permission.^[^ ^164^ ^]^ Copyright 2019, American Chemical Society. c) Self‐generated microfibrils network on a SBS block copolymer film and its use for the stretchable Au electrode made by vacuum deposition through a mask. Reproduced with permission.^[^ ^165^ ^]^ Copyright 2018, Wiley‐VCH. d) SBS block copolymer (BCP) film to fabricate a highly stretchable multilayer composite electrode. The microfibril network structure in the Ag flake/SBS composite layer maintained the electrical resistance between the composite layer and the liquid metal line the same under stretching. Reproduced with permission.^[^ ^166^ ^]^ Copyright 2019, American Chemical Society.

Adhesion between the layers is a key issue in highly stretchable devices. In order to maximize the adhesion between layers, structural design of the substrate surfaces has been attempted. For instance, Liu et al. presented that strong adhesion and stretchability were obtained by introducing the nanopile interlocking structure at the interface between the Au layer and the elastic substrate (**Figure** **13**a).^[^ ^162^ ^]^ The nanopile not only provided an interlocking effect that improved the adhesion but also redistributed and released the applied tensile strain. The adhesion of the Au film was significantly enhanced by more than ten times compared to the flat Au film, and the stretchability of the electrode reached *ε* = 40%. Similarly, Wu et al. demonstrated that an Ag layer plated on the biomimetic microporous elastomer substrate exhibited a strong adhesion (3.1 MPa) at the interface and showed a large tensile limit strain exceeding *ε* = 70%.^[^ ^163^ ^]^ The microporous structure regularly adjusted the strain distribution and prevented the formation of large cracks while being stretched. The microstructured interface was also used for making robust electrode/electrolyte interface in the stretchable supercapacitor. Fang et al. introduced microstructures between the tough hydrogel electrolyte and the activated carbon electrodes (Figure 13b).^[^ ^164^ ^]^ The microstructured interface improved the electrochemical performance and also the adhesion between the electrode and electrolyte, making the supercapacitor highly stretchable (*ε* = 150%) without performance degradation after repeated stretching cycles.

The microstructured interfaces were also used for developing stretchable conductors. Moon et al. created a microstructured network self‐generated on the thermoplastic substrate while the substrate was stretched (Figure 13c).^[^ ^165^ ^]^ When the SBS triblock copolymer film was elongated over *ε* = 100%, the crazes developed only on the surface. As the crazes continued to develop in many places they turned into a network of microfibrils. The network had microbridges interconnecting the microfibrils, thus the surface microstructure was biaxially stretchable. Direct sputtering of metals on the surface microfibril network allowed fabrication of highly stretchable conductors and strain sensors depending on the deposition thickness. The same group adopted microfibril networked SBS block copolymer film to fabricate a highly stretchable multilayer composite electrode (Figure 13d).^[^ ^166^ ^]^ The mesh type liquid metal line pattern was sandwiched between the Ag flakes/SBS microfibrilary composite (MFC) film and a SBS block copolymer (BCP) film. The composite electrode showed metallic conductivity (>10^4^ S cm^−1^), high stretchability (600%), and no resistance change (∆*R* < 0.04 Ω at *ε* = 300%). This excellent electrical stability could be achieved because the microfibril network structure in the Ag flake/SBS composite layer made the interfacial resistance between the composite layer and the liquid metal line the same under stretching.

### 4.3 Integration of Multiple Layers with Strong Interface

**Figure 14:**
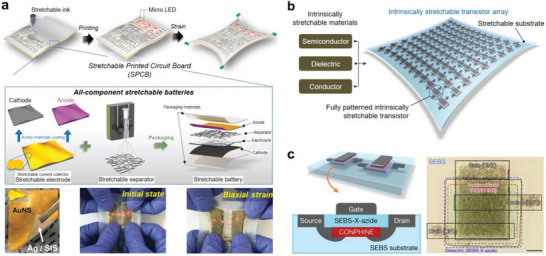
Intrinsically stretchable interfaces by all‐component stretchable multilayers. a) Intrinsically stretchable device platform by integrating a stretchable film battery and a stretchable printed circuit board. All the components in the battery were produced by the thermoplastic block copolymer composites so that the layers became unified without interfaces by mild thermal annealing. Reproduced with permission.^[^ ^167^ ^]^ Copyright 2020, Wiley‐VCH. b) Intrinsically stretchable polymer transistor array composed of intrinsically stretchable materials (dielectric, semiconductor, electrodes) prepared by inkjet printing. c) Structures and components of the transistor array where all transistor components are primarily based on the thermoplastic SEBS block copolymer. Reproduced with permission.^[^ ^18^ ^]^ Copyright 2018, Springer Nature.

Fabrication of stretchable devices using all the device components intrinsically stretchable has been explored a lot.^[^ ^18^, ^167^, ^168^ ^]^ In this approach, all the stretchable multilayers should have strong interfaces and the moduli of the layers should be comparable. Each stretchable layer can be prepared by the methods introduced in Sections 2 and 3. This approach has recently succeeded in the fabrication of simple stretchable devices such as batteries and transistors. Song et al. developed an intrinsically stretchable electronic device platform by integrating a stretchable film battery and a stretchable printed circuit board (SPCB) (**Figure** **14**a).^[^ ^167^ ^]^ The stretchable battery was fabricated as a substrate, and the SPCB was prepared by nozzle printing on the surface of the battery substrate, producing a stand‐alone electronic platform which was stably operated at *ε* = 100%. The stretchable battery components (current collector, anode, cathode, and separator) and the SPCB circuit lines were prepared in the composites of thermoplastic block copolymer elastomers (SBS and polystyene‐*block*‐polyisoprene‐*block*‐polystyrene (SIS)). Due to the viscoelastic adhesion and the thermoplastic deformability of the block copolymers, the interfaces between the multilayers were strongly adhered by low‐temperature thermal annealing, and the cuts of the SPCB circuit lines could be healed at mild temperature. Using the same thermoplastic adhesion strategy, Bao and co‐workers developed stretchable transistor arrays in which all the transistor components were intrinsically stretchable.^[^ ^18^, ^168^ ^]^ Wang et al. demonstrated an intrinsically stretchable polymer transistor array with a density of 347 transistors cm^−2^ (Figure 14b).^[^ ^18^ ^]^ All the components of the transistor array were primarily based on the thermoplastic SEBS block copolymer, so the interfacial delamination could be effectively prevented. They used SEBS as a substrate and crosslinkable SEBS‐X‐azide layer as the dielectric. The SEBS‐X‐azide dielectric enabled the solution‐processed printing of the channel layer which was the SEBS/conducting polymer composite layer (CONPHINE). The source/drain/gate electrodes were SEBS/CNT composites (Figure 14c). The transistors could operate at *ε* = 100% without defects (cracks, delaminations) and wrinkles. Molina‐Lopez et al. used SEBS as an encapsulating material and developed an inkjet‐printed stretchable synaptic transistor array by using intrinsically stretchable transistor components.^[^ ^168^ ^]^ They used materials with different mechanical properties such as ionic gel as dielectric, ionic PEODT:PSS as source/drain/gate electrodes, and semiconducting single‐wall carbon nanotubes (SC‐SWCNTs) as the active channels. All the components were inkjet‐printed and encapsulated by a thin film of SEBS. After encapsulation, they made the via holes by inkjet‐dropping toluene on the SEBS encapsulation layer and filled the holes with multiwall CNTs (MWCNTs). This process allowed easy interconnection of the transistors in the array. The SEBS encapsulation prevented the interfacial delamination and made the transistors operte under stretching (*ε* = 20%).

**Figure 15:**
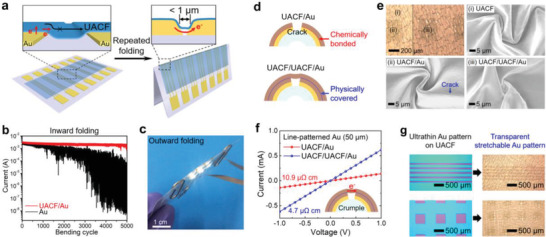
Deformable electrodes and interconnections by using the ultrathin anisotropic conductive film (UACF). a) Scheme illustrating the UACF coated on a flexible printed circuit board. The UACF is conductive in the thickness direction but shows limited conduction in the lateral direction, hence it can electrically connect a cracked metal line at the folded state. b) Current changes during repeated cycles of inward folding in the UACF/Au line (red) and pure Au line (black). c) Lighting of LEDs connected to the UACF/Au circuit board at the outward folded state. Reproduced with permission.^[^ ^169^ ^]^ Copyright 2019, American Chemical Society. d) Scheme describing the connection of the cracked region by the UACF cover layer in the UACF/UACF/Au electrode. e) OM and SEM images simultaneously showing the crumpled regions of UACF (i), UACF/Au (ii), and UACF/UACF/Au (iii). Surface cracks were formed along the folding lines only in the crumpled UACF/Au. f) Current–voltage curves and resistivity of the line‐patterned (50 µm in width) UACF/Au and UACF/UACF/Au stretchable electrodes. g) OM images showing the Au patterns formed on the UACF and the corresponding conversion to the transparent stretchable UACF/UACF/Au pattern. Reproduced with permission.^[^ ^170^ ^]^ Copyright 2020, American Chemical Society.

Very recently, a new approach for fabrication of high‐definition stretchable electrode and circuit line has been reported. It is based on the multilayer interface between metal thin film and the ultrathin anisotropic conductive film (UACF).^[^ ^169^, ^170^ ^]^ Pal et al. reported an easy method to produce the UACF which is an ultrathin hydrogenated amorphous carbon film (**Figure** **15**a).^[^ ^169^ ^]^ The UACF had conductance limitation in the lateral direction up to 1.6 µm, but it had no tunneling barrier in the thickness direction. When the UACF was transferred on a complicated metal circuit, the UACF/metal hybrid circuit could reflect the high‐resolution complexity and the high conductivity of the underlying metal circuit lines. When the metal circuit lines were cracked by repeated folding, the UACF could connect the cracks and maintain the electrical conduction of the metal circuit line, thus a completely foldable circuit was possible to produce (Figure 15b). This strategy provides a new way of fabricating a foldable paper‐like electronic devices, as demonstrated in Figure 15c. Kim et al. developed the concept further and succeeded in fabrication of transparent stretchable metal electrodes and high‐definition circuit lines.^[^ ^170^ ^]^ They used the UACF as a substrate for the deposition of a transparent ultrathin Au film to form a transparent UACF/Au circuit electrode and transferred another UACF layer onto the UACF/Au electrode (Figure 15d). The average on‐set strains of crack formation in the UACF and UACF/UACF bilayer were 2.35% and 2.41%, therefore cracks did not develop under harsh crumpling (Figure 15e). The on‐set strain in the UACF/Au bilayer electrode was 0.57% which was the same as that of an Au single layer, thus it cracked under folding and crumpling. This small on‐set strain of the UACF/Au electrode was because the stress of the high modulus Au was transmitted through the interface due to the strong interface by the chemical bonding. In the UACF/UACF/Au hybrid electrode, the weak interface between the two UACF layers blocked the stress transfer from the bottom UACF/Au layer, hence the top UACF layer did not crack under harsh crumpling and electrically connected over the cracks formed in the underlying UACF/Au layer (Figure 15f). Applying the prestrain process, the hybrid electrode attained biaxial stretchability (*ε* = 50%), and 50 µm‐wide stretchable Au circuit lines maintained the initial metallic conductivity under stretching (Figure 15g).

## 5 Design of Macroscale Interfaces

Integration of device components needs stable interfaces with mechanical and electrical durability. This macroscale interface has been overlooked in the stretchable electronics society. Device failure often occurs in the macroscale electrical interfaces, especially in the stretchable devices which has large mechanical deformations between the device components with different moduli. The primary interfaces are the connections between the stretchable circuit line and the electrode pads of the device components such as controller chips, sensors, LEDs, antenna for wireless communication, power source, external flexible printed circuit board, and so on.^[^ ^167^, ^171^ ^]^ The metal pads can be directly mounted on the stretchable circuit line, or can be connected through wires. Vertical connection between the circuit lines is also required in the multilayered device structures. The device has an interface with the object surfaces on which it is used. Many stretchable devices are to be used on the human skin, implanted inside a live animal, textiles, robotic arms, curved surfaces with dynamic deformation, and so on. Each object interface has unique requirements, thus the application‐driven interface designs are necessary.

### 5.1 Stable Electrical Connections

**Figure 16:**
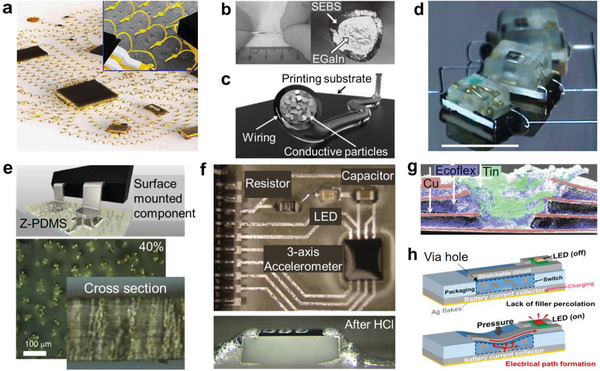
Various electrical interconnection methods. a) Stretchable 3D interconnection network of helical microcoils formed by compressive force of the prestrained metal lines. Reproduced with permission.^[^ ^104^ ^]^ Copyright 2017, Springer Nature. b) Stretchable metal wire composed of EGaIn core and SEBS shell. Reproduced with permission.^[^ ^121^ ^]^ Copyright 2013, Wiley‐VCH. c) Self‐wiring conductive wire consisting of Ag flakes and silicon rubber. Reproduced with permission.^[^ ^98^ ^]^ Copyright 2013, Wiley‐VCH. d) 3D printing of liquid metal wires to electrically connect μLEDs. Reproduced with permission.^[^ ^124^ ^]^ Copyright 2019, AAAS. e) Stretchable ACF made by vertically aligning Ag‐coated Ni microparticles in the PDMS matrix under magnetic field. Reproduced with permission.^[^ ^174^ ^]^ Copyright 2015, American Chemical Society. f) EGaIn soldering for chip bonding. Reproduced with permission.^[^ ^175^ ^]^ Copyright 2018, Wiley‐VCH. g) Via hole made in the silicon rubber filled with conductive metal solder paste (Sn_42_Bi_57.6_Ag_0.4_). Reproduced with permission.^[^ ^171^ ^]^ Copyright 2018, Springer Nature. h) Pressure responsive switch interconnection between the stretchable battery and the circuit board. Reproduced with permission.^[^ ^167^ ^]^ Copyright 2020, Wiley‐VCH.

Conventional electrical connections are often achieved with metal wires.^[^ ^95^, ^100^, ^124^, ^171^ ^]^ Nonstretchable curved metal wires have been used extensively by wire bonding. Jang et al. fabricated 3D interconnect networks of helical microcoils formed by the compressive process (**Figure** **16**a).^[^ ^104^ ^]^ The 3D helical structure offered an exceptionally low modulus and stretchability (up to *ε* = 300%) compared to the 2D serpentine structure. The 3D helical interconnection was fabricated by depositing serpentine structure metal ribbons (PI/Au/PI) over a prestrained elastomer substrate. Strong covalent siloxane bonds (>8 J m^−2^) were formed only in the localized points with ozonated hydroxyl groups. The out‐of‐plane rotational 3D buckling structure was generated at the nonadhesive area when the prestrain was released. Zhu et al. reported a stretchable metal wire made of the EGaIn liquid metal (Figure 16b).^[^ ^121^ ^]^ They fabricated liquid metal fibers by filling EGaIn in hollow SEBS fibers fabricated by the melt drawing process. Liquid metal could be injected into the hollow fibers when the external pressure exceeded the pressure required to rupture the oxide skin. Once EGaIn was injected in the SEBS fiber, new oxide skin was formed. The core–shell fiber could be massively produced. The fiber maintained the metallic conductivity even when elongated over *ε* = 800%. Yoon et al. demonstrated 3D‐printed wires consisting of Ag flake and silicone rubber (Figure 16c).^[^ ^98^ ^]^ When the printed wire was annealed at 100 °C for 4 h, the Ag flake was self‐concentrated in the center and the silicone rubber was at the surface of the fiber due to surface energy difference between the Ag flakes (1.2 J m^−2^) and silicon rubber (27 mJ m^−2^). This phase separation‐induced wire was stretchable up to *ε* = 113% and had a moderate electrical conductivity (85.91 S cm^−1^). Park et al. 3D‐printed EGaIn wires to electrically connect μLEDs (Figure 16d).^[^ ^124^ ^]^ 3D printing of EGaIn lines was possible due to the rapid formation of the oxide skin. The EGaIn wires were metallic conductive with a large current density (≈10^10^ A m^−2^) before electrical breakdown. The wires could be encapsulated with PDMS for enhanced passivation and stretchability.

Direct chip bonding is preferred for device reliability and high‐density integration. The anisotropic conductive film (ACF) which is conductive in the thickness direction but insulating in the lateral direction has been commonly used for surface mounted chip bonding.^[^ ^172^, ^173^ ^]^ Lu et al. reported a stretchable ACF by vertically aligning the Ag‐coated Ni MPs (15 µm in diameter) in the PDMS rubber matrix under magnetic field (Figure 16e).^[^ ^174^ ^]^ The alignment of the microparticles had an average separation of 50–75 µm, thus the film was nonconductive in the lateral direction (lateral capacitance 1.29–1.85 pF) and conductive only in vertical direction. The vertical resistance was varied according to the mass fraction of the conductive particles, film thickness, and the measurement area. At a fixed mass fraction (40%) and a thickness (69 µm), the vertical resistance decreased from 2.0 to 0.2 Ω when the area was increased from 1 to 9 mm^2^. The separation was maintained without delamination while the ACF was stretched up to *ε* = 30%. Ozutemiz et al. reported EGaIn soldering for chip integration (Figure 16f).^[^ ^175^ ^]^ As liquid metal thin films dewet on most surfaces due to their high surface energy, obtaining good wettability was a key to fine patterning. They used the patterns of Cr and Cu as a wetting layer for liquid metal. Liquid metal quickly formed a metal alloy which made a stable interface and prevented dewetting. After placing LED on the liquid metal pattern, they exposed HCl vapor to etch the oxide skin so that liquid metal could solder the LED terminals.

Monolithic vertical stacking increases device integration density and accommodates the state‐of‐the‐art circuit components and functions.^[^ ^167^, ^171^ ^]^ Electrical connection between the layers is usually performed through via hole. Huang et al. generated via holes (100 µm in depth, 45 µm in diameter) in the silicon rubber (Ecoflex) by the laser ablation and filled the hole with conductive metal solder paste (Sn_42_Bi_57.6_Ag_0.4_) (Figure 16g).^[^ ^171^ ^]^ Molina‐Lopez et al. made via holes using pure solvent to dissolve polymer layer and filled them by inkjet‐printing with a composite of CNTs and polyacrylonitrile (PAN).^[^ ^176^ ^]^ Hydrophobic PAN protected the water penetration. Song et al. fabricated a stand‐alone stretchable electronic device platform powered by stretchable rechargeable battery.^[^ ^167^ ^]^ They made a pressure‐responsive switch by filling the via hole with conductive block copolymer composite and connected the stretchable battery to the circuit board (Figure 16h). They controlled the Ag flake mass fraction (SIS:Ag = 1:3, w/w) to make the composite have an insulator‐to‐conductor transition (a few MΩ to 0.5 Ω) when a critical pressure was applied.

### 5.2 Skin Interface for Long‐Term Use

**Figure 17:**
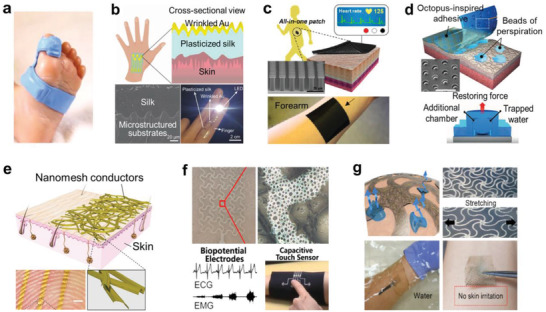
Skin‐attached stretchable substrates. a) Wireless skin‐attached soft biosensor using a low modulus silicon substrate as a biocompatible adhesive. Reproduced with permission.^[^ ^177^ ^]^ Copyright 2020, Springer Nature. b) Highly stretchable tough silk protein adhesive enabled by adding CaCl_2_ as a plasticizer. Reproduced with permission.^[^ ^178^ ^]^ Copyright 2018, Wiley‐VCH. c) Gecko‐inspired hierarchical PDMS micropillar structure as a dry adhesive patch. Reproduced with permission.^[^ ^179^ ^]^ Copyright 2016, American Chemical Society. d) Octopus‐inspired suction cups for wet‐tolerant adhesive patch. Reproduced with permission.^[^ ^180^ ^]^ Copyright 2017, Springer Nature. e) Nanomesh stretchable electrode attached directly on the skin without any substrate. Reproduced with permission.^[^ ^183^ ^]^ Copyright 2017, Springer Nature. f) Gas‐permeable, ultrathin, stretchable electrode made of porous thermoplastic polyurethane and Ag NWs. Reproduced with permission.^[^ ^184^ ^]^ Copyright 2020, American Chemical Society. g) Hygroscopic auxetic on‐skin sensor electrode using a serpentine‐structured PEGDA hydrogel. Reproduced with permission.^[^ ^185^ ^]^ Copyright 2018, American Chemical Society.

Primary application of the stretchable electronics is skin‐attached devices.^[^ ^177^, ^178^ ^]^ The skin interface requires specific properties on the device substrate: stable contact of the electrodes, controllable adhesion, breathability for sweat evaporation and oxygen penetration, and biaxial stretching. Although a practical substrate that meets all the requirements has not been achieved yet, there have been partial advances on the substrates and the number of related publications are increasing. Chung et al. fabricated a practical wireless skin‐attached soft biosensor for physiological monitoring (**Figure** **17**a).^[^ ^177^ ^]^ The sensor could measure heart rate, respiration rate, temperature, and blood oxygenation. The sensor used a low modulus silicone (LMS) substrate which also plays as a long‐lasting biocompatible adhesive. Silk protein is one of the promising skin interface material due to its biocompatibility and biodegradability. However, silk has high Young's modulus (5–12 GPa) and low stretchability (<20%) which limits its wide applications. Chen et al. produced a silk protein substrate by adding CaCl_2_ as a plasticizer (Figure 17b).^[^ ^178^ ^]^ Ca^2+^ ions rendered the moisture into silk fibroin from ambient environment, thus its Young's modulus decreased considerably to 0.1–2 MPa and its stretchability increased to *ε* > 400%. Highly stretchable electrodes could be fabricated by thin‐film metallization of wrinkled silk surface. Plasticized silk electrode was attached conformably on a finger skin and could operate an LED.

Many researches have been investigated to increase skin adhesion by constructing bioinspired structures on the substrate.^[^ ^179^, ^180^, ^181^, ^182^ ^]^ A few representative studies are introduced in this review. Kim et al. demonstrated enhanced adhesion by constructing gecko‐inspired hierarchical PDMS micropillar structures (Figure 17c).^[^ ^179^ ^]^ Each micropillar had a spatula tip head (5–7 µm in diameter) and long thin pillar body (10–20 µm in length) connected to the substrate. A long pillar with a wider spatula tip was appropriate for attaining large van der Waals adhesion. A silicon mold pattern of negative straight holes with a round end was fabricated on the Si wafer by deep reactive ion etching (DRIE). The positive pillars were created by pouring PDMS prepolymer solution in the mold pattern, followed by thermal curing at 120 °C. Normal adhesion force of the mushroom‐shaped pillar pattern was 1.3 N cm^−2^ even on the rough human skin. The pattern maintained the same adhesion during 30 times cycling test. The modulus of the micropillars had a trade‐off relationship between adhesion and mechanical endurance. Low‐modulus micropillars often caused mechanical failure during the demolding process and the collapse of the pillars or mating between neighboring pillars. On the other hand, high‐modulus micropillars decreased the adhesion force. Baik et al. fabricated octopus‐inspired suction cups for wet‐tolerant adhesive patch (Figure 17d).^[^ ^180^ ^]^ The dome‐like protuberance of the suction cup was made of polyurethane‐acrylate polymer (PUA) with 50 µm radius dome structure. Liquid precursor of PUA was partially filled in the microscale holes in the silicon mold. The partial filling was based on force balance between the capillary rise of liquid precursor and the pressure by the trapped air bubbles in the microhole. Mechanical adhesion was achieved by three steps: volume decrease of the octopus‐inspired architecture (OIA) chamber by normal pressure, squeeze of the OIA and liquid trap at the chamber top, and creation of vacuum chamber at the bottom after removing the normal pressure. Normal adhesion force increased when exposed to wet or oily conditions due to its high viscosity (37 kPa in moist, 41 kPa under water, 154 kPa under silicon oil). It maintained good adhesion to various substrates under both wet and dry conditions even after 10 000 cycles of attachment test. Choi et al. applied this suction structure to a smart medical patch.^[^ ^181^ ^]^ They integrated physiological sensors, drug delivery actuators, and therapeutic NPs. The bioinspired structure minimized the tissue damage and inflammation. Lee et al. used the octopus sucker structure to demonstrate a smart hydrogel adhesive with thermo‐responsive actuation.^[^ ^182^ ^]^ By using PNIPAM which undergoes a phase transition at a lower critical solution temperature (LCST), they controlled the volume change of the PNIPAM for actuation. The adhesion could be controlled in the range of 1.0–59.1 kPa when the temperature was increased from 22 to 35 °C.

Breathability of the substrate is critical for long‐term skin attachment (more than several hours) without inflammation of the skin. Miyamoto et al. demonstrated nanomesh stretchable electronics mounted on the skin without any substrate (Figure 17e).^[^ ^183^ ^]^ The metal nanomesh electrode was fabricated by depositing gold on an electrospun PVA nanofiber mat and dissolving PVA by water. The thin metal nanomesh made conformal contacts on the irregular surfaces of the skin and showed excellent mechanical durability under strain. However, repeated use of a device requires a substrate which a user can handle manually. Zhou et al. presented gas‐permeable, stretchable electrode using porous thermoplastic polyurethane (TPU) and Ag NWs (Figure 17f).^[^ ^184^ ^]^ The porous TPU film was prepared by the breath figure method to have the pore diameter of 40 µm and pore coverage of about 39%. Ag NWs were hot pressed over this porous TPU to be embedded in the polymer surface. The thickness of the hot‐pressed thin film was 4.6 µm which enabled conformal contact with the skin. The sheet resistance of the film was 7.3 Ω sq^−1^ and the water evaporation rate was 23 mg cm^−2^ h^−1^. The film could be stretched up to *ε* = 15%. When the film was stretched first, its resistance increases by 4–7 times, but after a few cycles the resistance change stabilizes to less than 7%. They applied this electrode in wireless human–computer interface as capacitive touch sensor. Porous hydrogel substrates are advantageous in the aspects that they can make a hygroscopic conformal interface. Kim et al. fabricated a hygroscopic auxetic on‐skin sensor electrode by using serpentine‐structured poly(ethyleneglycol)diacrylate (PEGDA) hydrogel (Figure 17g).^[^ ^185^ ^]^ The auxetic structure with a negative Poisson's ratio followed the biaxial deformation of the human skin, maintaining the conformal contact with the skin under severe body motions. Sweat evaporated through the empty space among the serpentine structure and also was absorbed to the hydrogel pattern and then evaporated quickly. This hygroscopic property maintained the contact to the skin. The electrode was stretchable up to *ε* = 30% without resistance change. The ECG sensor could operate safely even in salt water, and no skin irritation or inflammation occurred while wearing for 7 d continuously.

### 5.3 Interface for Implantable Devices

**Figure 18:**
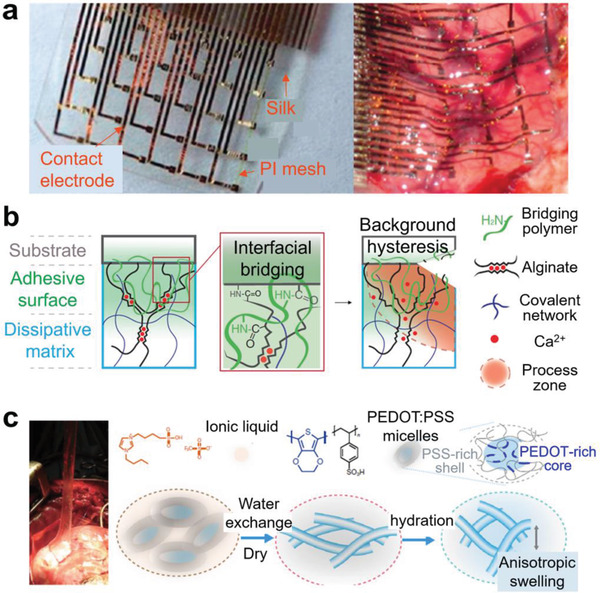
Interface engineering for implantable devices. a) Ultrathin electronics supported by bioresorbable silk nanofiber substrate. Reproduced with permission.^[^ ^186^ ^]^ Copyright 2019, Springer Nature. b) Strong implantable adhesive composed of an energy dissipative hydrogel matrix and a reactive surface layer. Reproduced with permission.^[^ ^125^ ^]^ Copyright 2017, AAAS. c) Conductive PEDOT:PSS based hydrogel for tissue. Reproduced with permission.^[^ ^187^ ^]^ Copyright 2019, Springer Nature.

Implantable devices are also attracting great attention in the field of soft electronics.^[^ ^125^, ^186^, ^187^ ^]^ In addition to the modulus mismatch with the internal organs and biocompatibility, the electrical interface is a great challenge in the implantable devices. The efforts placed so far have been mainly on mechanical engineering with biocompatible materials. Kim et al. fabricated ultrathin electronics supported by bioresorbable silk fibroin substrate (**Figure** **18**a).^[^ ^186^ ^]^ After fabricating a metal electrode on the surface of PMMA/PI, PMMA was dissolved, and the electrode was transferred to an ultrathin silk film (2.5 µm). The electrode array was implanted on the brain, and the silk substrate was dissolved by flushing with saline. The electrode made conformal contact on 3D‐shaped organs. Lee et al. fabricated an active multielectrode array (MEA) for long‐term electrocardiography (ECG) monitoring. They chose 100 nm thick poly(3‐methoxypropyl acrylate) (PMC3A) for antithrombotic property and for uniform conformal contact. A honeycomb grid structure made of the substrate and PEDOT:PSS was used for mechanical stability and stretchability. After coating with PMC3A, transconductance of the MEA was stable for 10 h.

Material design and chemical reaction were also explored to enhance the interface. Li et al. reported a strong adhesive bilayer composed of an energy dissipative matrix and a reactive surface layer (Figure 18b).^[^ ^125^ ^]^ The adhesive was bound to various substrates through electrostatic interactions, covalent bonds, and physical interactions, whereas the tough matrix dissipated energy by hysteresis under deformation. Alginate‐polyacrylamide (Alg‐PAAm) hydrogel was used for the energy dissipating substrate. The adhesive surface layer was made of polyallylamine (PAA) with positively charged primary amine functional groups. PAA could be absorbed to the tissue via electrostatic attractions and covalent bonds with amine groups of the tissue. Strong adhesion force could be achieved (>1116 J m^−2^) even under blood exposure. Water permeability of the bilayer was higher than the normal skin or muscles. Liu et al. fabricated a conductive PEDOT:PSS‐based soft hydrogel (32 kPa) (Figure 18c).^[^ ^187^ ^]^ It showed a low interfacial impedance with the tissue, a high current‐injection density larger than that of the platinum electrode, and stable electrical performance under large strains. The same group reported that addition of ionic liquid in PEDOT:PSS solution promoted the formation of long PEDOT nanofibers and increased the conductivity.^[^ ^73^ ^]^ After creating the PEDOT:PSS network gel in the presence of the ionic liquid, the hydrogel was cleaned by exchanging the ionic liquid by water. Surprisingly, the ionic conductance remained the same after exchanging by water. The soft hydrogel was attached stably at the outer surface of the sciatic nerve and had conformal contacts.

## 6 Perspectives and Challenges

**Figure 19:**
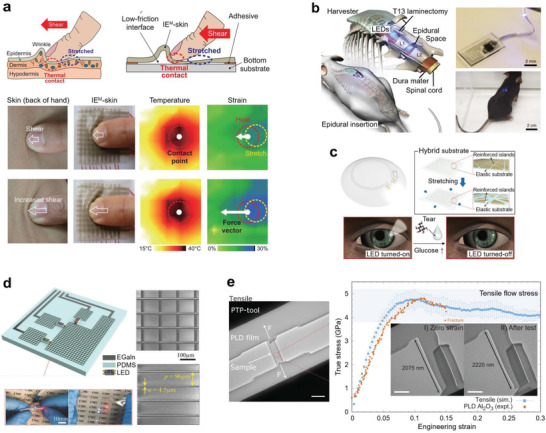
Perspectives and technological challenges in stretchable electronics. a) Human skin‐like multimodal ion‐electronic skin (IE^M^‐skin) for independent simultaneous measurement of temperature and strain. The sensor was wrinkled by strain as the human skin does, and the force directions and strain profiles are obtained from the information of temperature and strain. Reproduced with permission.^[^ ^188^ ^]^ Copyright 2020, AAAS. b) Implantable and stretchable optogenetic neural interface system to stimulate spinal pain nerves. Reproduced with permission.^[^ ^41^ ^]^ Copyright 2015, Springer Nature. c) Stretchable and transparent smart contact lens for detecting a glucose level in tear. Reproduced with permission.^[^ ^189^ ^]^ Copyright 2018, AAAS. d) Stretchable transparent liquid metal circuitry with high definition. Reproduced with permission.^[^ ^193^ ^]^ Copyright 2018, Wiley‐VCH. e) Measurement of tensile stress of the amorphous Al_2_O_3_ film according to tensile strain. The amorphous Al_2_O_3_ shows the plastic deformation with a yield strain of about 10%, which is unusual and not fully investigated. Reproduced with permission.^[^ ^197^ ^]^ Copyright 2019, AAAS.

As electronic devices with new form factors such as “foldable” and “rollable” are commercialized, interest in stretchable devices is increasing. Although a lot of researches have been carried out on stretchable materials and devices, it is still necessary to solve many technological issues for commercialization. As the technologies used in the conventional rigid electronic devices will be modified to fit the soft devices in the early stage of commercialization, paying attention to the technological trends in the conventional devices can give hints to the future research directions of stretchable devices. Current electronic devices evolve toward miniaturization, multifunctionality, and systems‐on‐a‐body. The miniaturization of stretchable devices requires significant advances in high‐definition circuit lines, high‐resolution electrical connections, and chip bonding to an operating microprocessor. A new design for small‐sized antenna for wireless communication is also necessary. Multifunctional devices are sought by either integrating different devices on one substrate or by obtaining multimodality in one device. Distinguishing various physical stimulations (pressure, strain, torque, temperature, etc.) can be the multifunctions in the stretchable sensors (**Figure** **19**a).^[^ ^27^, ^28^, ^29^, ^30^, ^31^, ^32^, ^188^ ^]^ Many combinations were reported to sense various stimuli (proximity, chemicals, magnetic field, etc.) and have the additional functions (display, actuation, etc.).^[^ ^33^, ^34^, ^35^, ^36^, ^37^, ^38^, ^39^, ^40^, ^41^ ^]^ In addition, by introducing a deformable supercapacitor or a stretchable battery, it is developing into a fully deformable stand‐alone device.^[^ ^167^ ^]^ The trend of systems‐on‐a‐body is one of the main target of the skin‐attached and implantable devices. Researches to mimic human sensory functions or to replace sensory organs have been steadily performed over the past half century. Wearable artificial aids such as hearing aids and contact lenses, and also cochlear implants, artificial retinas, and artificial nerve systems have made great technological progresses (Figure 19b,c).^[^ ^39^, ^41^, ^189^, ^190^ ^]^ Stretchable tactile sensors have proved wide applicability in haptic devices, wearable healthcare sensors, electronic skin for robots, prosthetics, and implanted medical devices such as cardiovascular or bladder sensors and electrical stimulators.^[^ ^27^, ^28^, ^29^, ^30^, ^31^, ^32^, ^33^, ^34^, ^35^, ^36^, ^37^, ^190^ ^]^ Production of commercial products of such devices will be the next step to be achieved in the stretchable electronics society.

Because the stretchable devices should maintain the high device performance during repeated folding and stretching events, securing stable interfaces is of particular importance. The molecules, nanostructures, microstructures, multiple layers, and unit devices forming the interfaces in stretchable devices have different modulus and mechanical hysteresis, therefore the interfaces need to be systematically studied according to the proper physical scales. The organic–organic composites with the nano‐ and microscale interfaces have focused on stretchability and self‐healing,^[^ ^58^, ^59^, ^63^ ^]^ without considering integration with other constituent elements. In order for the organic materials to be used in practical unit devices or in integrated device arrays, the process stability (high‐temperature processing, film uniformity, material compatibility to other units, etc.) and the environmental stability (moisture, solvents, temperature variation, etc.) should be secured. Currently, high‐performance devices are made with inorganic materials (conductors, semiconductors, and dielectrics) or their polymer composites.^[^ ^97^, ^98^, ^99^, ^100^, ^101^, ^102^, ^103^, ^104^, ^105^, ^106^, ^107^, ^108^, ^109^, ^110^, ^111^, ^112^, ^113^, ^114^, ^115^, ^116^, ^117^, ^118^, ^119^, ^120^ ^]^ In the nano‐ and microscale interfaces of the inorganic–organic composites, the stress distribution depending on the direction, size, and shape of the inorganic materials are in lack of knowledge. In addition, there has been little studies on the fatigue of those interfaces caused by the friction during repeated mechanical deformations. In the mesoscale interfaces, minimizing the strain effect is the key technology. The buckling and rigid islands approaches can be readily applied to low‐resolution devices.^[^ ^142^, ^143^, ^144^, ^145^, ^146^, ^147^, ^148^, ^149^, ^150^, ^151^, ^152^, ^153^, ^154^, ^155^, ^156^, ^157^, ^158^, ^159^, ^160^, ^161^ ^]^ On the other hand, the intrinsically stretchable device approach may provide high integration, but the stretchable functional materials are still far behind their practicality. Therefore, there is a need for a new approach that can overcome simultaneously the issues of the high‐resolution integration and the lack of materials. In the macroscale interfaces, high‐resolution stretchable electrical connections are utmost necessary between stretchable circuit lines and the metal pads of the microchips or the flexible printed circuit boards (FPCBs). For instance, the brain–computer interfaces requesting high‐density circuit lines and miniaturized operating chips need a technology to electrically connect the circuit lines with less than 100 µm width.^[^ ^191^ ^]^ Although many skin‐attachable devices are being developed, little attention has been paid to substrates. The substrates for long‐term uses on the skin interface should be biocompatible and water permeable. Nonpermeable substrates such as PDMS might cause skin irritation when attached more than 2–3 h.^[^ ^192^ ^]^ The water‐permeable substrates with good adhesion and high stretchability are prone to both the skin‐attached biosensors and the implantable sensors in terms of stable electrode–tissue interface.

Currently, the biggest obstacle to highly integrated stretchable devices is a lack of high‐definition stretchable circuit lines. Although many stretchable conductors have been reported so far, practical fabrication of the circuit lines having the conductivity and pattern resolution (less than 10 µm line width) comparable to conventional metal lines has not yet been achieved. Stretchable transparent electrodes also have been investigated for displays, solar cells, and touch screen panels.^[^ ^79^, ^170^ ^]^ Unfortunately, transparent electrodes satisfying the reasonable stretchability, high conductivity, and micropatterning have not yet been studied intensively (Figure 19d).^[^ ^193^ ^]^ Graphene and Ag NWs were suggested as transparent stretchable electrodes, but their conductivity decreased and the electrical resistance varied significantly under stretching when they are micropatterned.^[^ ^194^ ^]^ The absence of an antioxidant stretchable packaging material is also a major problem. A multilayer structure of an oxide thin film and a polymer thin film are being used as a packaging layer in organic light‐emitting diodes.^[^ ^195^, ^196^ ^]^ A similar structure may be used for stretchable packaging film, but not successful so far. The lack of those materials mentioned above is largely due to the limitations of the material properties. Attempts to overcome the conventional concepts for material properties may open the window to find new materials. Recently, a study revealed that an amorphous Al_2_O_3_ thin film can be viscoplastic so that it can be stretched without crack formation (Figure 19e).^[^ ^197^ ^]^ This viscoplasticity of an amorphous oxide gives the possibility of producing inorganic semiconductors and dielectrics that are deformable up to a large degree of tensile strains. Material design and structural optimization on the basis of artificial intelligence will be of great help in improving the compatibility between the materials, overcoming the limits of material properties, and predicting the characteristics of the devices.

## Conflict of Interest

The authors declare no conflict of interest.
